# Protective effects of *Ganoderma lucidum* spores on estradiol benzoate-induced TEC apoptosis and compromised double-positive thymocyte development

**DOI:** 10.3389/fphar.2024.1419881

**Published:** 2024-08-16

**Authors:** Jihong Yang, Haitao Pan, Mengyao Wang, Anyao Li, Guoliang Zhang, Xiaohui Fan, Zhenhao Li

**Affiliations:** ^1^ College of Pharmaceutical Sciences, Zhejiang University, Hangzhou, Zhejiang, China; ^2^ BoYu Intelligent Health Innovation Laboratory, Hangzhou, Zhejiang, China; ^3^ ShouXianGu Botanical Drug Institute, Hangzhou, Zhejiang, China; ^4^ National Key Laboratory of Chinese Medicine Modernization, Innovation Center of Yangtze River Delta, Zhejiang University, Jiaxing, Zhejiang, China

**Keywords:** *Ganoderma lucidum* spores, T cell development, thymic atrophy, T cell receptor gene rearrangement, single-cell RNA sequencing, proteomics

## Abstract

**Backgroud:** Thymic atrophy marks the onset of immune aging, precipitating developmental anomalies in T cells. Numerous clinical and preclinical investigations have underscored the regulatory role of *Ganoderma lucidum* spores (GLS) in T cell development. However, the precise mechanisms underlying this regulation remain elusive.

**Methods:** In this study, a mice model of estradiol benzoate (EB)-induced thymic atrophy was constructed, and the improvement effect of GLS on thymic atrophy was evaluated. Then, we employs multi-omics techniques to elucidate how GLS modulates T cell development amidst EB-induced thymic atrophy in mice.

**Results:** GLS effectively mitigates EB-induced thymic damage by attenuating apoptotic thymic epithelial cells (TECs) and enhancing the output of CD4^+^ T cells into peripheral blood. During thymic T cell development, sporoderm-removed GLS (RGLS) promotes T cell receptor (TCR) α rearrangement by augmenting V-J fragment rearrangement frequency and efficiency. Notably, biased Vα14-Jα18 rearrangement fosters double-positive (DP) to invariant natural killer T (iNKT) cell differentiation, partially contingent on RGLS-mediated restriction of peptide-major histocompatibility complex I (pMHCⅠ)-CD8 interaction and augmented CD1d expression in DP thymocytes, thereby promoting DP to CD4^+^ iNKT cell development. Furthermore, RGLS amplifies interaction between a DP subpopulation, termed DPsel-7, and plasmacytoid dendritic cells (pDCs), likely facilitating the subsequent development of double-negative iNKT1 cells. Lastly, RGLS suppresses EB-induced upregulation of Abpob and Apoa4, curbing the clearance of CD4^+^Abpob^+^ and CD4^+^Apoa4^+^ T cells by mTECs, resulting in enhanced CD4^+^ T cell output.

**Discussion:** These findings indicate that the RGLS effectively mitigates EB-induced TEC apoptosis and compromised double-positive thymocyte development. These insights into RGLS’s immunoregulatory role pave the way for its potential as a T-cell regeneration inducer.

## 1 Introduction

Thymic atrophy leads to developmental disorders and reduced output of T cells, which is the main reason for the decline of immune function and the increasing incidence of infectious diseases and cancer (Palmer et al., 2018; Lian et al., 2020). Functional T cells develop through a series of processes, which initiates with double-negative (DN) precursors, lacking both CD4 and CD8 expression, then undergo T cell receptor (TCR) β gene rearrangement to become double-positive (DP, CD4^+^CD8^+^) thymocytes. Following TCRα rearrangement, DP thymocytes undergo positive selection by recognizing peptide-major histocompatibility complex (pMHC) presented by cortical thymic epithelial cells (cTECs), leading to the development of single positive (SP, CD4^+^CD8^−^ or CD4^−^CD8^+^) thymocytes (Vacchio et al., 2016). Only DP thymocytes with specific TCR affinity acquire MHC restriction, differentiating into SP thymocytes (Vacchio et al., 2016). Approximately 80% of DP thymocytes undergo programmed cell death due to a lack of appropriate pMHC signals (Li et al., 2021b). Subsequently, SP thymocytes undergo screening by medullary thymic epithelial cells (mTECs) to achieve central tolerance, ultimately becoming naïve T cells that migrate to peripheral tissues to execute immunomodulatory functions (Li et al., 2021b). Additionally, invariant natural killer T (iNKT) cells, a distinctive subset of innate-like T cells expressing both T cell and NK cell receptors, include iNKT1 (NK1.1^+^), iNKT2 (NK1.1^−^), and iNKT17 (NK1.1^−^) subtypes (Bennstein, 2017). Originating from common lymphoid progenitors, iNKT cells, during the DP phase, recognize glycolipid antigens presented by CD1d on DP thymocytes, deviating from conventional TEC-dependent selection (Sahler et al., 2021). iNKT cells activate various immune cells by secreting cytokines like interferon-γ (IFN-γ) and interleukin-2 (IL-2) (Nelson et al., 2021) to exert diverse biological functions, including anti-tumor, anti-viral, anti-obesity, and anti-diabetes activities (Magalhaes et al., 2015; Bonamichi and Lee, 2017; Cui et al., 2022). Efforts to stimulate their expansion *in vivo* have shown promise, albeit with challenges (Liu et al., 2022b). However, systematic elucidation of thymic iNKT cell development remains a considerable challenge, hampering the discovery of effective therapies.

Estrogens such as estradiol impair the development of thymic T cells and lead to hypofunction of the thymus (Rijhsinghani et al., 1996). Sex hormone blockade therapy is considered an effective strategy to reverse thymic atrophy (Velardi et al., 2015). However, the blockade of sex hormones can cause significant side effects such as cognitive decline, immune decline, and muscle atrophy (de Souza Santos et al., 2017; Moulton, 2018; Gurvich et al., 2021). Furthermore, there is still no standard therapy to enhance T cell regeneration in thymic atrophy. Therefore, drugs are urgently needed to target T cell development to relieve thymic hypofunction caused by sex hormones effectively.

*Ganoderma lucidum* (*G. lucidum*), a medicinal mushroom with a historical usage spanning over 6,800 years in China (Yuan et al., 2018), produces minute reproductive cells known as G. lucidum spores (GLS). These spores, encapsulated in a resilient chitin shell sporoderm, contain active ingredients such as polysaccharides and triterpenes (Liu et al., 2020). Sporoderm-removing methods enhance the bioavailability and bioactivity of GLS by eliminating the chitin barrier (Chen et al., 2024). Our prior analyses systematically explored the chemical profiles of GLSs produced via different manufacturing processes, elucidating that sporoderm-removed GLS (RGLS) contains a higher abundance and diversity of active ingredients compared to sporoderm-broken GLS (BGLS) (Li et al., 2020). Furthermore, RGLS exhibited a favorable safety profile in preclinical evaluation studies (Xia et al., 2023) and demonstrated superior efficacy in anti-tumor and immune regulation when compared to BGLS (Li et al., 2020; Fang et al., 2022). Despite the established T cell-mediated immunomodulatory effects of GLS, current studies focus on peripheral blood T cells, leaving a significant gap in understanding the regulation of T cell development in the thymus and its impact on iNKT cell differentiation.

In this study, we induced thymic atrophy in mice using estradiol benzoate (EB) to interfere with T cell development. Then, we evaluated the effects of BGLS and RGLS on EB-induced thymic atrophy and T-cell proportions in peripheral blood. Our findings highlight the significant potential of high-dose RGLS (RGLSH) in ameliorating thymic atrophy and enhancing CD4^+^ T cell proportions. Subsequent molecular analyses revealed that RGLSH promotes thymic epithelial cell (TEC) proliferation by inhibiting cell cycle arrest and apoptosis. Furthermore, our study integrated genomic, transcriptomic, and proteomic to elucidate the impact of RGLSH on thymocyte subtypes, demonstrating an increase in DP thymocytes and a decrease in DN thymocytes. Mechanistically, RGLSH regulates the development of DP thymocytes into DP-selected iNKT cells by promoting Vα14-Jα18 TCRα chain formation.

Additionally, RGLSH influences the differentiation of DP-selected iNKT cells into CD4^+^ iNKT1 cells by modulating pMHC expression, increasing CD1d expression, and inhibiting pMHC-CD8 interaction in DP thymocytes. Moreover, RGLSH regulates the differentiation of DP-selected iNKT cells into DN iNKT1 cells by enhancing the CD226-CD155 interaction between DP-selected iNKT cells and plasmacytoid dendritic cells (pDCs). This comprehensive exploration of the molecular mechanisms underlying RGLSH’s impact on thymic development provides valuable insights into potential strategies for enhancing iNKT cell therapy.

## 2 Results

### 2.1 GLS alleviates EB-induced impairment of thymic T cell development

To assess the effects of BGLS and RGLS on EB-induced thymic atrophy, we conducted efficacy evaluation experiments *in vivo* (Supplementary Figure S1A). There were no significant changes in the body weight of mice across all experimental groups (Supplementary Figure S1B). Nevertheless, EB administration resulted in a noteworthy reduction in both thymic weight and thymic index, and this impact was not reversed by either Ube, BGLS, or RGLS (Supplementary Figure S1C). However, it remained unclear whether the pathological changes and internal morphological structure of the thymus were affected. Subsequently, we conducted a pathological analysis within the thymus tissue using H and E staining. As shown in Supplementary Figure S1D, the EB-treated group exhibited a distinct blurring of the boundary between the cortex and medulla of thymus tissue, resulting in a thinner cortical area. Conversely, the Ube and GLS treatment groups displayed a well-defined demarcation between the cortex and medulla, accompanied by a thickened cortical area, closely resembling the morphological characteristics of thymus tissue observed in the control group. Intriguingly, our observations also revealed an increased density of thymic cortical regions in the EB-treated group compared to the Con group, while Ube, BGLS, and RGLS exhibited a mitigating effect on this phenomenon (Supplementary Figure S1D).

Following the histological assessments, we proceeded to analyze the apoptosis of thymic epithelial cells (TECs) through TUNEL staining. As demonstrated in Supplementary Figure S1E, the proportion of apoptotic cells (indicated in brown) within thymus tissue exhibited a significant increase in the EB-treated group. Conversely, Ube, BGLS, and RGLS demonstrated a notable reduction in EB-induced cell apoptosis (Supplementary Figure S1F). Next, we assessed white blood cell (WBC) counts in the peripheral blood and measured the proportion of T cells within these WBCs. As depicted in Supplementary Figure S1G, EB treatment resulted in a significant decrease in total WBC counts, while Ube, BGLS, and RGLS exhibited the ability to increase total WBC counts. Notably, the proportion of T cells within total WBCs did not exhibit significant changes in any group (Supplementary Figure S1H), indicating a rescue of total T cell number by BGLS and RGLS. Furthermore, we investigated the proportions of specific T cell subtypes, including CD3^+^CD4^+^ and CD3^+^CD8^+^ T cells, relative to total T cells. EB treatment significantly reduced the proportion of CD4^+^ T cells, whereas both Ube and high-dose RGLS (RGLSH) significantly increased the proportion of CD4^+^ T cells. Additionally, no significant difference was observed in the proportion of CD8^+^ T cells among groups (Supplementary Figures S2A, B). These results indicate that GLS mitigates EB-induced impairment of thymic T cell development and increases the proportion of CD4^+^ T cells in peripheral blood, with RGLS showing a more pronounced effect than BGLS.

### 2.2 RGLSH inhibits EB-induced TECs apoptosis

**FIGURE 1:**
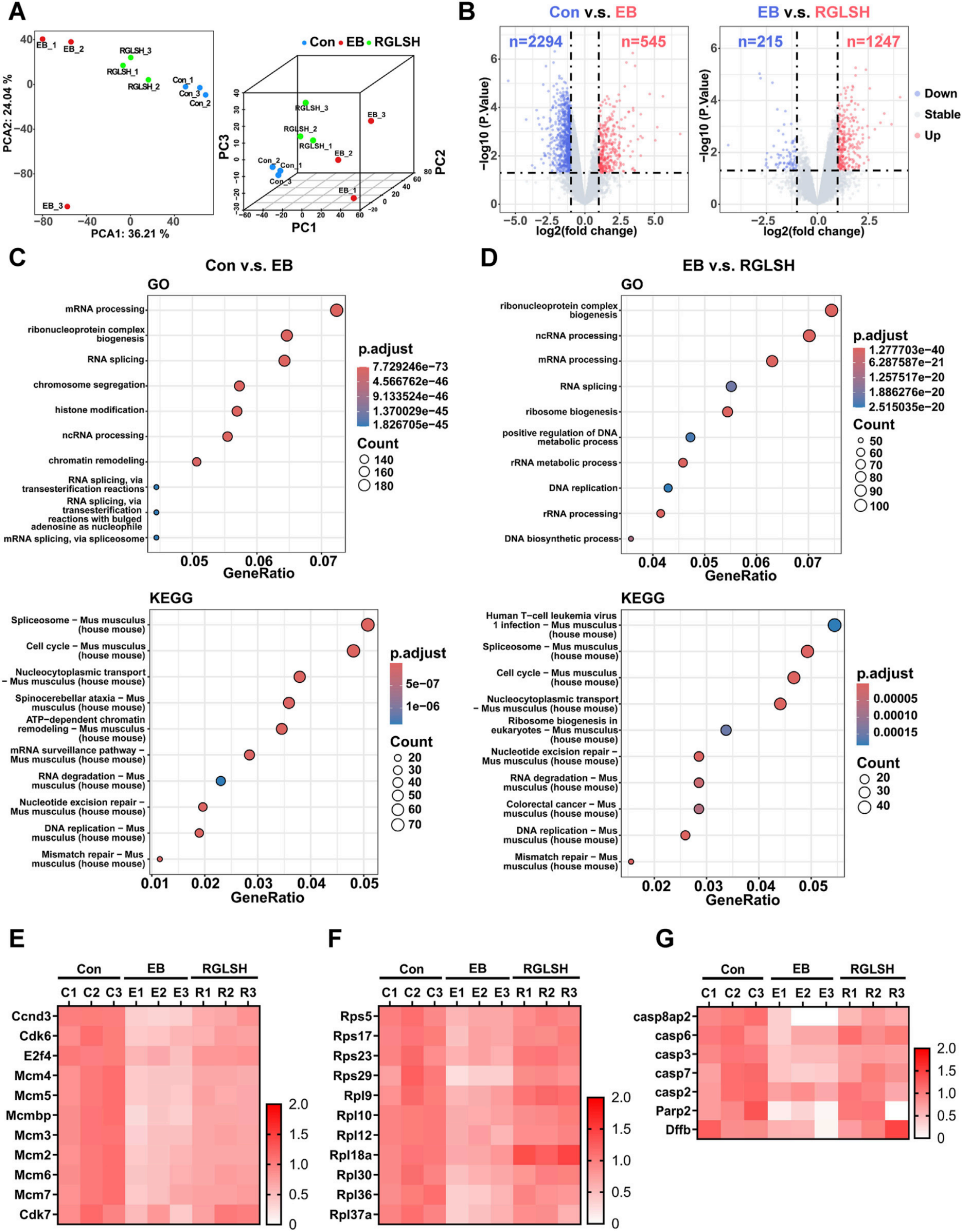
Proteomics analysis of thymus tissues. **(A)** Principal component analysis (PCA) of proteomic data. **(B)** Differential expression analysis of proteomic data. **(C,D)** Gene Ontology (GO) and Kyoto Encyclopedia of Genes and Genomes (KEGG) terms enriched by differentially expressed proteins. **(E)** Effect of RGLSH treatment on the expression of cell cycle-related proteins in the thymus tissue. **(F)** Effect of RGLSH treatment on the expression of ribonucleoproteins in the thymus tissue. **(G)** Effect of RGLSH treatment on the expression of apoptosis-related proteins in the thymus tissue.

The inhibitory effect of estrogen on thymic epithelial cell (TEC) proliferation, through the induction of cell apoptosis, is a pivotal factor in estrogen-induced thymic atrophy (Wei et al., 2018; Liu et al., 2022a). Building upon the above findings, we sought to unravel the molecular mechanisms underlying the regulatory role of RGLSH in compromising EB-induced TECs apoptosis. RGLSH did not alleviate the EB-induced decrease in thymic weight and index (Supplementary Figure S2C). Then, we employed proteomics to analyze protein changes in thymus tissues. Principal component analysis (PCA) revealed significant alterations in protein expression profiles of TECs induced by EB and RGLSH compared to the Con group (Figure 1A). Specifically, compared to the Con group, EB significantly upregulated 545 proteins and downregulated 2294 proteins. In contrast, RGLSH, when compared to the EB group, upregulated 1247 proteins and downregulated 215 proteins (|log2 (fold change)| ≥ 1 and p-value <0.05) (Figure 1B). Furthermore, gene ontology (GO) enrichment analysis highlighted the involvement of the upregulated or downregulated proteins in processes such as ribonucleoprotein complex biogenesis, mRNA processing, RNA splicing, chromatin remodeling, ribosome biogenesis, DNA replication, and double-strand break repair. Kyoto Encyclopedia of Genes and Genomes (KEGG) analysis revealed significant alterations in the expression levels of the spliceosome and cell cycle-related proteins (Figures 1C, D). Gene set enrichment analysis (GSEA) further confirmed significant changes in cell cycle, RNA splicing, chromatin remodeling, ribosome biogenesis, DNA replication, and repair-related genes (Supplementary Figures S3A–F). These analyses suggest that RGLSH may enhance protein synthesis, RNA splicing, and DNA replication and repair in TECs, thereby promoting cell proliferation and survival. Indeed, our analysis showed a series of proteins, demonstrating that RGLSH significantly counteracted the EB-induced downregulation of cell cycle-related proteins (including the G1/S checkpoints Ccnd3, Cdk6, E2f4, and G2/M checkpoints Cdk7) (Figure 1E) and ribonucleoproteins (Rps5, Rps17, and Rps23) (Figure 1F). Additionally, RGLSH significantly downregulated the apoptosis-related proteins (including the Casp family, Parp2, and Dffb) induced by EB (Figure 1G).

**FIGURE 2:**
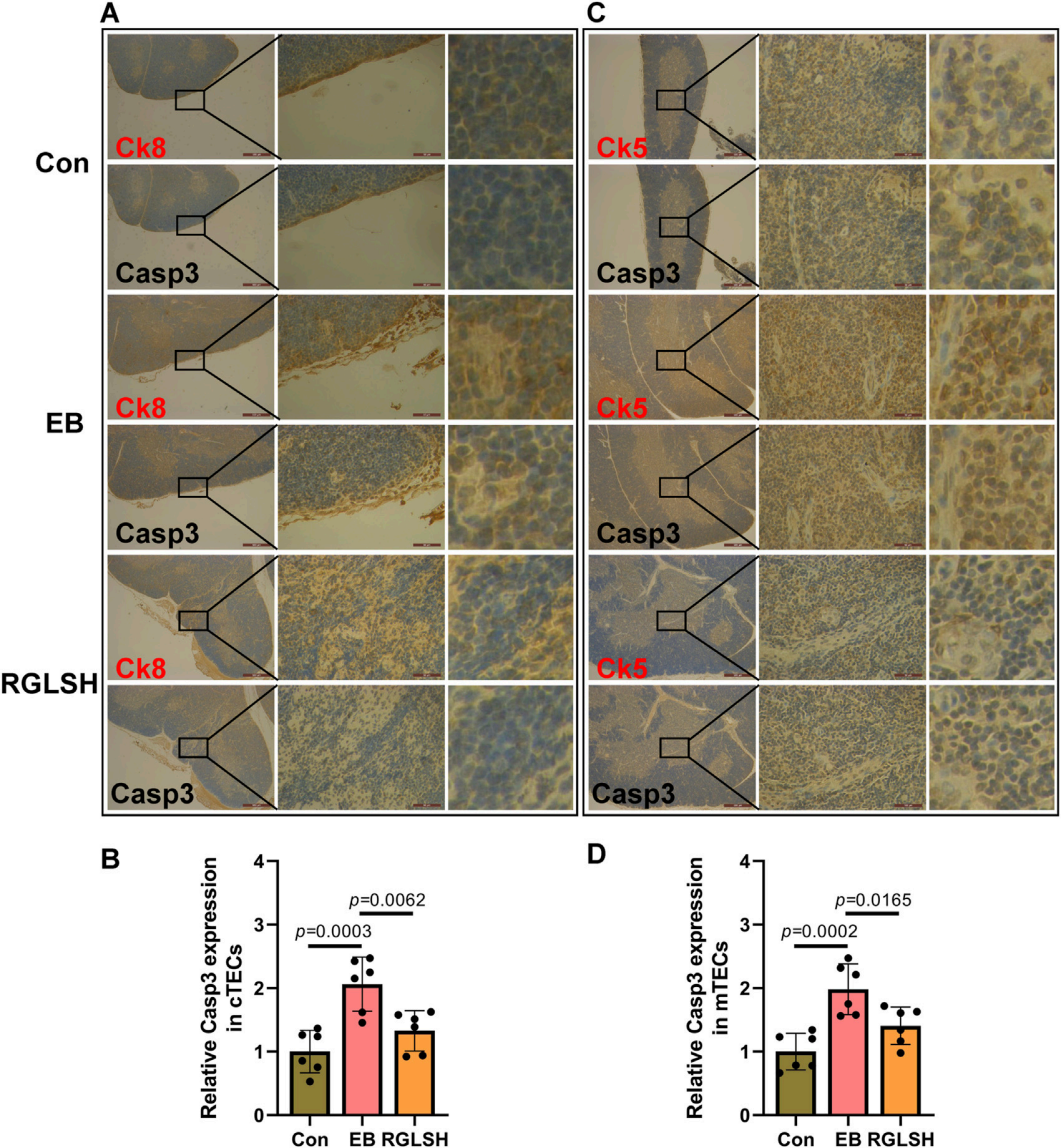
The co-localization of cytokeratin (CK) and Casp3 were analyzed using immunohistochemistry (IHC) staining in thymus tissues. **(A,B)** The co-localization of CK8 and Casp3 **(A)** and relative quantitative analysis **(B)**. **(C,D)** The co-localization of CK5 and Casp3 **(C)** and relative quantitative analysis **(D)**. Scale bar = 500 μm (40 ×), 50 μm (400 ×), n = 6.

The structure of thymus is complicated, in order to better reflect the protective effect of RGLSH on the apoptosis of TECs, the co-localized immunohistochemistry (IHC) was performed to detect the effect of RGLSH on the expression level of Casp3 protein in CK8^+^ TECs (cTECs) and CK5^+^ TECs (mTECs), and the results were shown in Figure 2. Compared with the Con group, EB treatment significantly increased the expression levels of Casp3 protein in CK8^+^ cTECs (Figures 2A, B) and CK5^+^ mTECs (Figures 2C, D). Conversely, RGLSH treatment obviously decreased Casp3 protein expression levels in CK8^+^ cTECs (Figures 2A, B) and CK5^+^ mTECs (Figures 2C, D) compared with the EB treatment group. These results collectively indicate that RGLSH ameliorates EB-induced TEC apoptosis.

### 2.3 RGLSH facilitates thymocyte development from DN to DP stage

Peripheral T cells predominantly originate from the thymus, a pivotal site for thymocyte development. Thymic atrophy often leads to aberrant thymocyte development (Heng et al., 2010). Generally, thymocyte progenitor cells undergo four stages, namely double negative (DN, CD4^−^CD8^−^), immature single positive (ISP, CD4^+^CD8^−^Mki67^+^ or CD4^−^CD8^+^Mki67^+^), double positive (DP, CD4^+^CD8^+^), and single positive (SP, CD4^+^ or CD8^+^) (Sawicka et al., 2014).

To assess the impact of RGLSH on alleviating EB-induced thymic atrophy on thymocyte development, we analyzed the proportion of thymocytes at different stages using flow cytometry. As depicted in Supplementary Figure S2D, E, EB treatment significantly increased the proportion of DN thymocytes and decreased the proportion of DP thymocytes in the thymus, indicating that EB inhibited thymocyte development from DN to DP stage. In contrast, RGLSH treatment significantly reduced DN but did not significantly increase DP proportion (Supplementary Figure S2D, E). This suggests that RGLSH fosters thymocyte development from the DN to the DP stage, and the DP thymocytes might further mature into later-stage cells, rather than stagnating in the DP stage.

### 2.4 RGLSH enhances DP rearrangement

**FIGURE 3:**
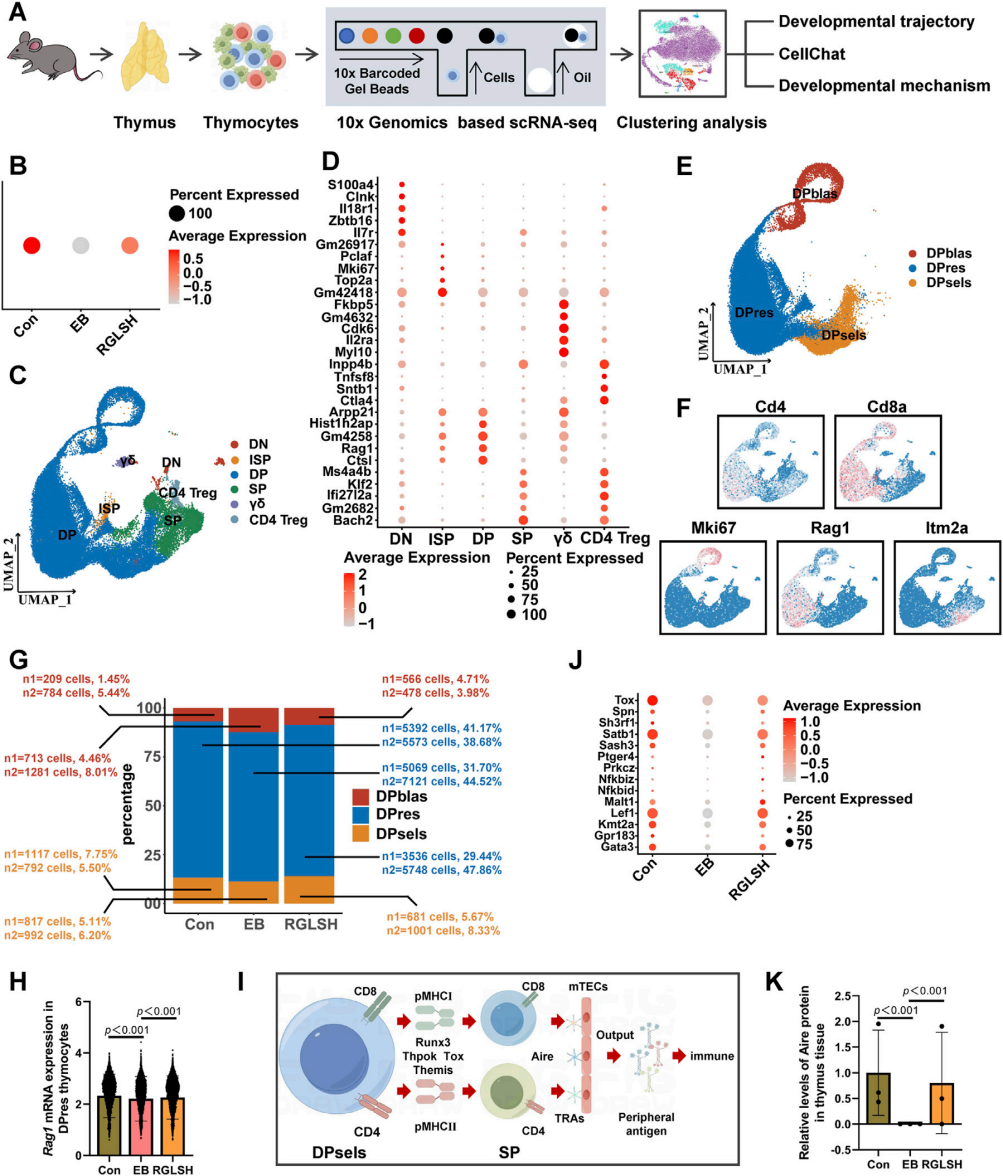
Single-cell RNA sequencing analysis of thymocytes. **(A)** Flowchart of single-cell RNA sequencing. **(B)** Gene signature analysis of T cell differentiation in thymus. **(C)** Cluster analysis of thymocytes and visualization in a two-dimensional uniform manifold approximation and projection (UMAP). **(D)** Cell-type-specific marker gene expression. **(E)** Cluster analysis of DP thymocytes and visualization in a two-dimensional UMAP. **(F)** CD4, CD8a, Mki67, Rag1, and Itm2a marker genes projected onto UMAP plots. **(G)** Effect of RGLSH treatment on the number of DPblas, DPres, and DPsels thymocytes and their proportion to DP thymocytes. **(H)** Effect of RGLSH treatment on the expression of *Rag1* mRNA in DPres thymocytes. **(I)** Schematic diagram of the developmental pattern of DPsels thymocytes. **(J)** Gene signature analysis of CD4^+^ T cell differentiation in thymus. **(K)** Effect of RGLSH treatment on the expression of Aire protein in the thymus tissue.

To gain deeper insights into the impact of RGLSH on thymocyte development, we employed an oil droplet-based single-cell RNA sequencing (scRNA-seq) platform (Figure 3A). Utilizing Seurat’s unsupervised analysis, cells were clustered into five groups and visualized in a two-dimensional uniform manifold approximation and projection (UMAP) (Supplementary Figure S4A). Notably, neither EB nor RGLSH treatments significantly alter the proportion of thymocytes (T cell lineage) (Supplementary Figure S4B), consistent with the observation that RGLSH did not affect the proportion of T cells in peripheral blood (Supplementary Figure S1H). Subsequently, gene signature analysis revealed that RGLSH promoted T cell development in the thymus (Figure 3B).

**FIGURE 4:**
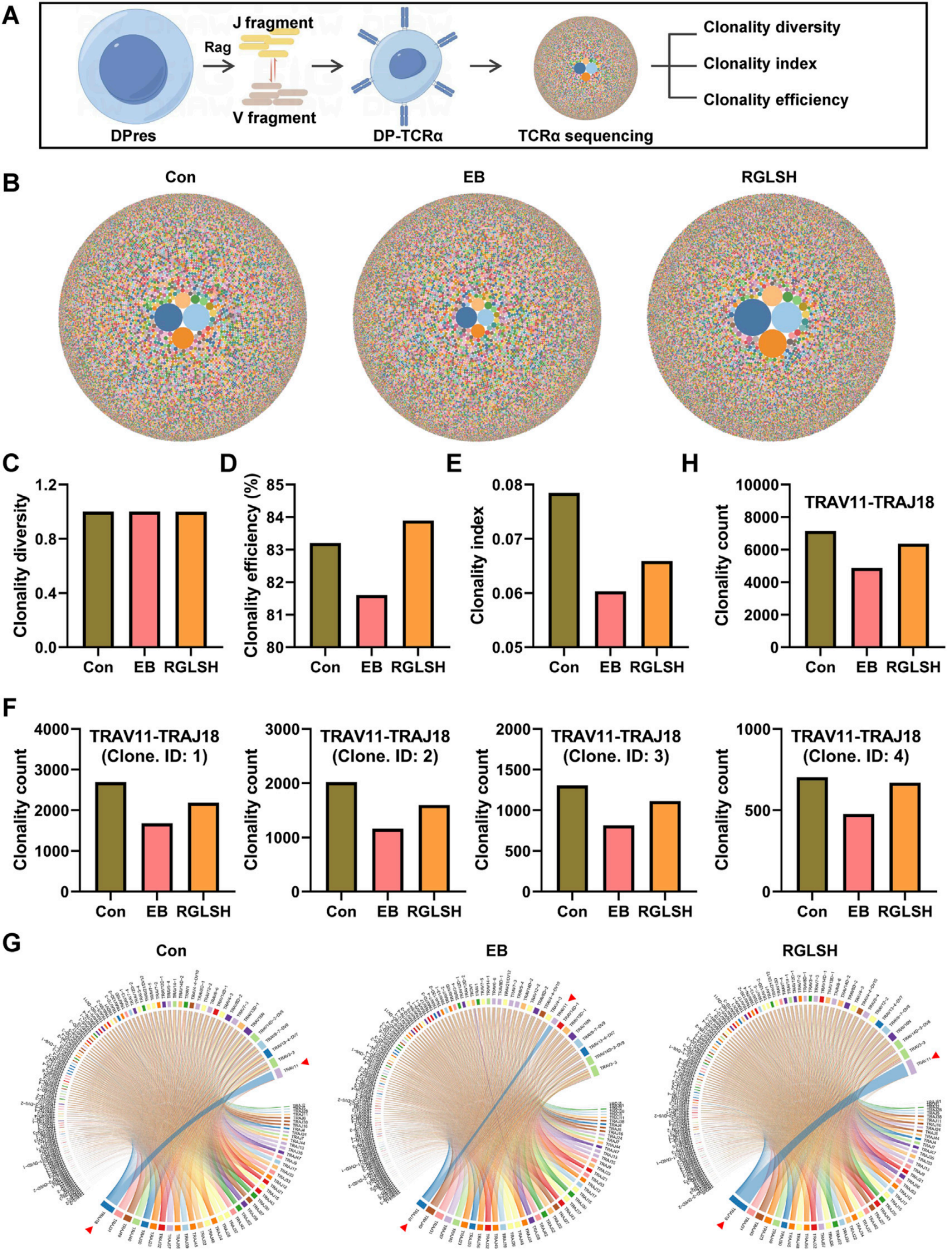
TCRα sequencing analysis of thymocytes. **(A)** Schematic diagram of the TCRα sequencing. **(B)** Bubble map of TCRα clone. **(C–E)** Effect of RGLSH treatment on the clonality diversity **(C)**, clonality efficiency **(D)**, and clonality index **(E)** of TCRα. **(F)** Effect of RGLSH treatment on the combination of different sequences TRAV11 and TRAJ18. **(G)** Circos map of TCR V fragment and J fragment combination. **(H)** Effect of RGLSH treatment on the total combination of TRAV11 and TRAJ18.

Aligned with previous flow cytometry results (Supplementary Figures S2D, E), RGLSH treatment changed the proportions of DN and DP thymocytes (Figures 3C, D). Furthermore, DP thymocytes were further classified into three sub-types based upon developmental progression from early to late stage (Li et al., 2021b): DP under blasts proliferation (DPblas, DP Mki67^+^), DP under rearrangement (DPres, DP Rag1high), and DP undergoing selection (DPsels, DP Itm2a^+^) (Figures 3E, F). Quantitative analysis revealed that RGLSH treatment increased the proportion of DPsels while decreasing the proportion of DPblas (Figure 3G), suggesting that RGLSH has the potential to convert more DPblas into DPsels by improving the efficiency of DP rearrangement. Subsequently, we investigated the effects of RGLSH on the expression of key rearrangement regulators Rag1 and Rag2. RGLSH significantly attenuated the inhibitory effect of EB on *Rag1* (Figure 3H), while havin g no impact on *Rag2* (Supplementary Figure S4C). Considering that the differentiation of DPres thymocytes is fundamentally a process in which V-J TCR fragments rearrange to form a mature TCRα chain (Pasetto and Buggert, 2022). To comprehensively analyze the impact of RGLSH on TCRα chain formation in DPres thymocytes, TCRα sequencing was performed (Figure 4A). As depicted in Figures 4B, C, neither EB nor RGLSH affected the diversity of the TCRα chain genes. However, RGLSH enhanced the clonality index (Figure 4D) and efficiency (Figure 4E). These results collectively suggest that RGLSH promotes the DPblas-DPres-DPsels developmental axis of thymocytes.

### 2.5 RGLSH modulates DP thymocyte selection and tolerance

Following the initiation of the selection phase, DP thymocytes recognize peptide-major histocompatibility complex (pMHC) presented by cortical thymic epithelial cells (cTECs), undergoing differentiation into CD4^+^ or CD8^+^ SP thymocytes (termed positive selection). Subsequently, SP thymocytes recognize tissue-restricted antigens (TRAs) presented by medullary TECs (mTECs) to achieve central tolerance (negative selection). Ultimately, SP thymocytes migrate out of the thymus to perform immunomodulatory functions after obtaining peripheral tolerance (Figure 3I).

As illustrated in Figure 3J, we performed gene signature analysis and screened several transcription factors that establish CD4^+^ T cell lineage, including Tox, Satb1, Sash3, Lef1, and Gata3, which are downregulated by EB but upregulated by RGLSH. The autoimmune regulator (Aire), a key tolerogenic transcription factor, regulates the expression of various tissue-restricted antigens (TRAs) in medullary thymic epithelial cells (mTECs) and establishes central tolerance by eliminating autoreactive thymocytes (Shevyrev et al., 2022). EB significantly reduced Aire expression, whereas RGLSH upregulated it (Figure 3K).

**FIGURE 5:**
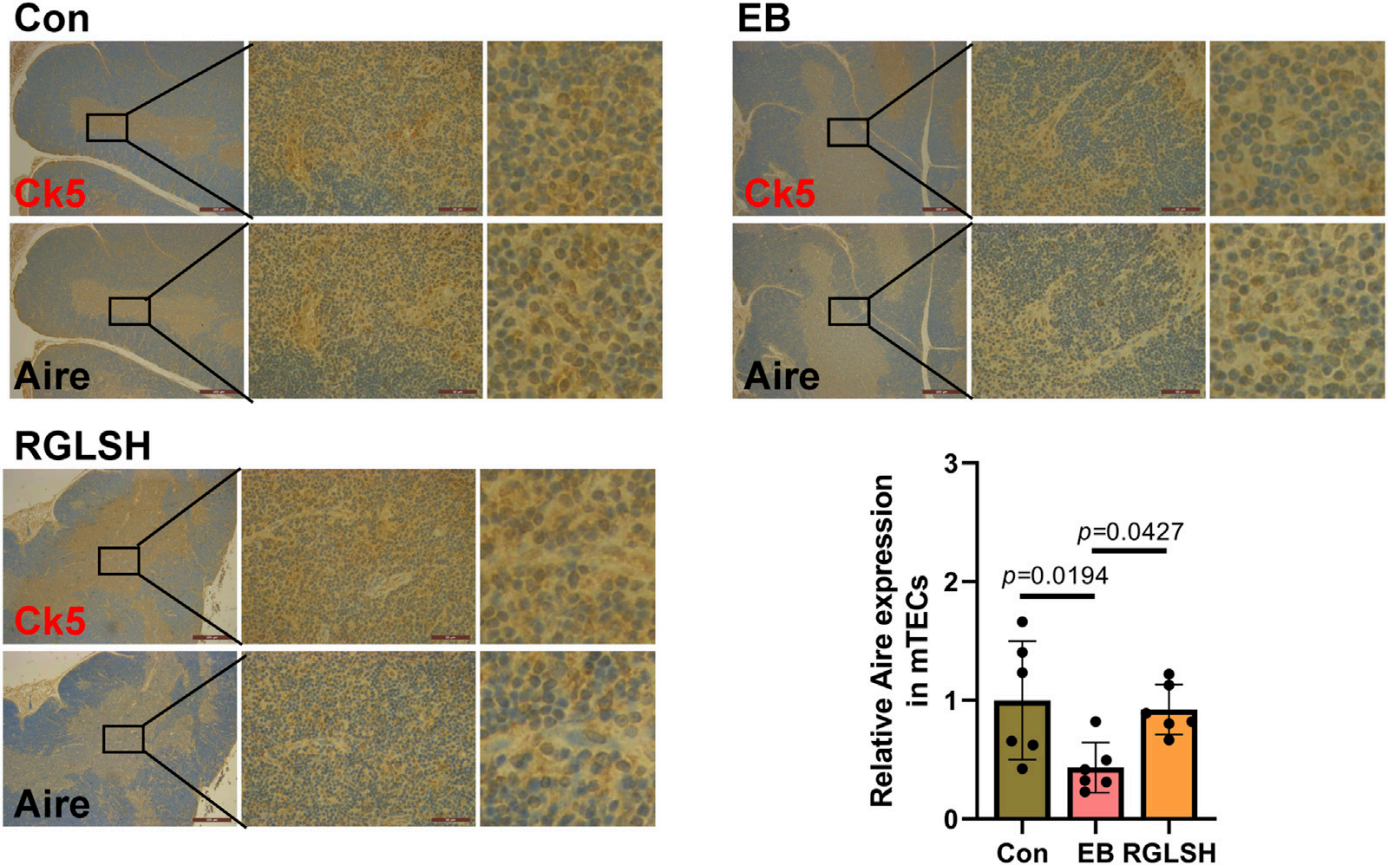
The co-localization of CK5 and Aire were analyzed using IHC staining in thymus tissues, and relative quantitative analysis. Scale bar = 500 μm (40 ×), 50 μm (400 ×), n = 6.

Further, in order to more accurately analyze the effect of RGLSH on Aire expression levels in mTECs, we performed co-localized IHC analysis of CK5 and Aire in thymus tissue. The result is shown in Figure 5. Compared with the Con group, EB treatment significantly decreased the expression levels of Aire protein in CK5^+^ mTECs. However, compared with the EB treatment group, RGLSH treatment obviously increased Aire protein expression levels in CK5^+^ mTECs. These results suggest that RGLSH promotes negative selection of thymocytes by up-regulating the expression of Aire in mTECs.

### 2.6 RGLSH facilitates the differentiation of DPres thymocytes into iNKT cells

Although RGLSH tends to develop DP thymocytes into CD4^+^ T cells, the exact development line is not clear due to the diversity of T cells. Moreover, both EB and RGLSH did not alter the expression of *pMHCⅡ* mRNA in thymus tissue (Supplementary Figure S4D), nor the expression of Thpok (encoded by *Zbtb7b*) (Supplementary Figure S4E), a transcription factor that regulate the development of DP thymocytes into CD4^+^ SP thymocytes (Overgaard et al., 2015), in Itm2a^+^ DP thymocytes. To understand the specific trends of DP cell development, we identified the TCRα chains influenced by RGLSH (Supplemental File S1), focusing on the top five based on the clonality index. Notably, the top five were centered on the TRAV11-TRAJ18 rearrangement (Figure 4F). Further analysis, with a specific focus on TRAV11-TRAJ18 rearrangement, revealed that RGLSH attenuated the inhibitory effect of EB on the combination of TCRα fragments TRAV11 and TRAJ18 (Figures 4G, H), encompassing combination frequency and usage frequency. Previous studies have established that TRAV11-TRAJ18 (Vα14-Jα18) TCRα chains are consistently expressed in the invariant natural killer T cells (iNKT) (Ren et al., 2018). This suggests that RGLSH may regulate a certain proportion of DPres thymocytes to enter the DPsels stage, eventually differentiating into iNKT cells.

**FIGURE 6:**
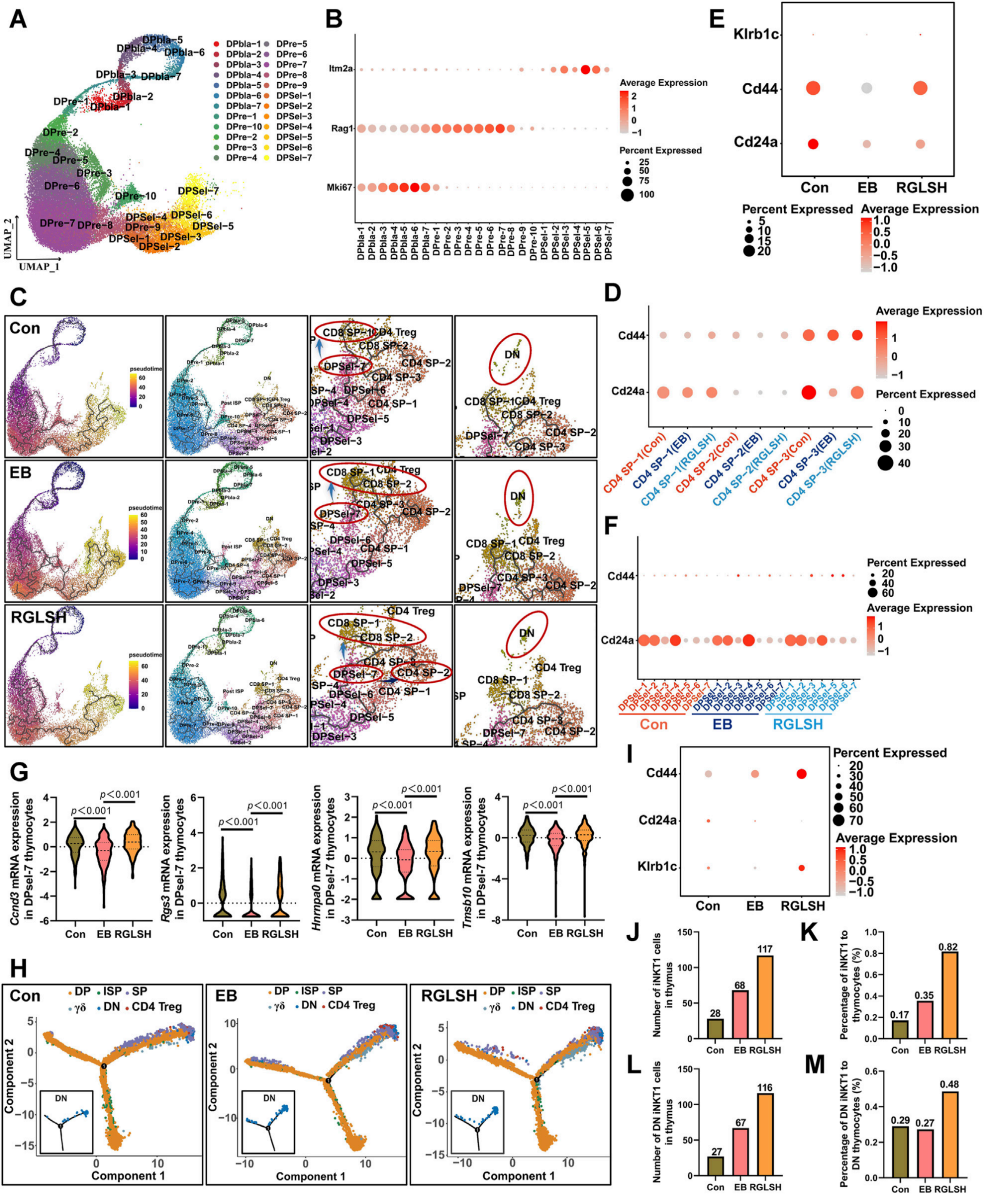
RGLSH facilitates the differentiation of DPsel-7 thymocytes into CD4^+^ iNKT cells. **(A)** Cluster analysis of DP thymocytes and visualization in a two-dimensional UMAP. **(B)** Expression levels of Mki67, Rag1, and Itm2a in different cell populations. **(C)** Effect of RGLSH treatment on the differentiation trajectories of thymocytes (Monocle3). **(D)** Effect of RGLSH treatment on the expression of CD44 and CD24a in CD4 SP cells. **(E)** Effect of RGLSH treatment on the expression of CD44, CD24a, and Klrb1c in CD4 SP-2 cells. **(F)** Effect of RGLSH treatment on the expression of CD44 and CD24a in DPsels thymocytes. **(G)** Effect of RGLSH treatment on the expression of cell cycle-related regulator in DPsel-7 thymocytes. **(H)** Effect of RGLSH treatment on the differentiation trajectories of thymocytes (Monocle2). **(I)** Effect of RGLSH treatment on the expression of CD44, CD24a, and Klrb1c in DN cells. **(J,L)** Effect of RGLSH treatment on the number of iNKT1 cells **(J)** and DN iNKT1 cells **(L)** in the thymus tissues. **(K,M)** Effect of RGLSH treatment on the proportion of iNKT1 cells **(K)** and DN iNKT1 cells **(M)** to thymocytes.

To further clarify the developmental trajectory, we divided DP thymocytes into 24 sub-groups based on the specific markers of each cell population (Figures 6A, B) and predicted the differentiation trajectories of thymocytes. As depicted in Figure 6C, during the continuous differentiation process of DPsels, DPsel-7 thymocytes exhibited distinct trajectories. Compared to that in the EB group, RGLSH treatment exhibited a stronger differentiation trend of driving DPsel-7 into CD4^+^ SP thymocytes. In mice, there mainly exist two iNKT sub-types, namely, CD4^+^ iNKT and DN (CD4^−^CD8^−^) iNKT (Rossi et al., 2012). We analyzed CD24a and CD44, two iNKT cells marker (Dinh et al., 2021), in three CD4 SP populations, namely, CD4 SP-1, SP-2, and SP-3, and found that only CD4 SP-2 aligned with the characteristic of iNKT cells (CD4^+^CD24a^low^CD44^high^, Figure 6D) (Dinh et al., 2021). RGLSH treatment resulted in higher CD44 expression compared to the EB group (Figure 6E). Moreover, approximately 2% CD4 SP-2 cells expressed NK1.1 (encoded by Klrb1c) in the RGLSH treatment group (Figure 6E), indicating that these iNKT cells were type 1 iNKT (iNKT1, CD4^+^CD24a^low^CD44^high^NK1.1^+^) (Bennstein, 2017). However, further investigation is needed to determine if RGLSH affects the characteristics and proportions of other cells, such as type 2 iNKT cells (iNKT2) and type 17 iNKT cells (iNKT17). The iNKT1 cells are developed from DPsel thymocytes (DPsel iNKT, namely stage 0 iNKT, CD24ahighCD44low) (Dinh et al., 2021). Furthermore, the expression trends of CD24a and CD44 in DPsel 1-7 showed that the expression of CD44 gradually increased along the development trajectory of DPsel-1→PDsel-4→DPsel-5→DPsel-6, and finally decreased at the DPsel-7 stage. It is suggested that DPsel-7 represents the terminal stage of DPsel iNKT, in which marker expression was consistent with the characteristic of the stage 0 iNKT (Figure 6F). The rapid proliferation is another characteristic of the stage 0 iNKT cells (Dinh et al., 2021). Consistently, RGLSH enhanced expression levels of several cell cycle-related genes, including Ccnd3, Hnrnpa0, Rgs3, and Tmsb10 (Figure 6G).

Stage 0 invariant natural killer T (iNKT) cells undergo differentiation, branching into both CD4^+^ and DN iNKT subtypes (Rossi et al., 2012). We further analyzed whether RGLSH regulates the formation of DN iNKT cells. Coincidentally, differentiation trajectories analysis based on Monocle2 and Monocle3 revealed a group of DN cells at the terminal stage of thymus cell development (Figures 6C, H), with approximately 50% of these DN cells expressing iNKT1 markers (CD24a^low^CD44^high^NK1.1^+^, Figure 6I). In mice, the development of iNKT1 cells represents only a minute fraction of thymocytes, constituting approximately 0.2%–0.46% of the thymic population (Zhang et al., 2015; Wang et al., 2019; Wang et al., 2022). Subsequently, we analyzed the number of iNKT1 cells and their proportion relative to thymocytes. As depicted in Figures 6J, K, RGLSH treatment significantly increased both the number of iNKT1 cells and their proportion in thymocytes compared to EB treatment. Interestingly, compared to the Con group, the number and proportion of iNKT1 cells also increased after EB treatment. This observation may be indicative of compensatory feedback regulation in response to EB-induced inhibition of NK1.1 expression, potentially compensating for iNKT1 cell dysfunction.

Specifically, the proportion of DN iNKT1 cells in DN cells exhibited a slight decrease compared to the control group, while RGLSH treatment, compared to EB treatment, increased the proportion of DN iNKT1 cells within DN thymocytes (Figures 6L, M). These results underscore RGLSH’s role in regulating the differentiation of DP thymocytes into iNKT1 cells.

### 2.7 RGLSH restricts pMHC-CD8 communication between DP thymocytes

**FIGURE 7:**
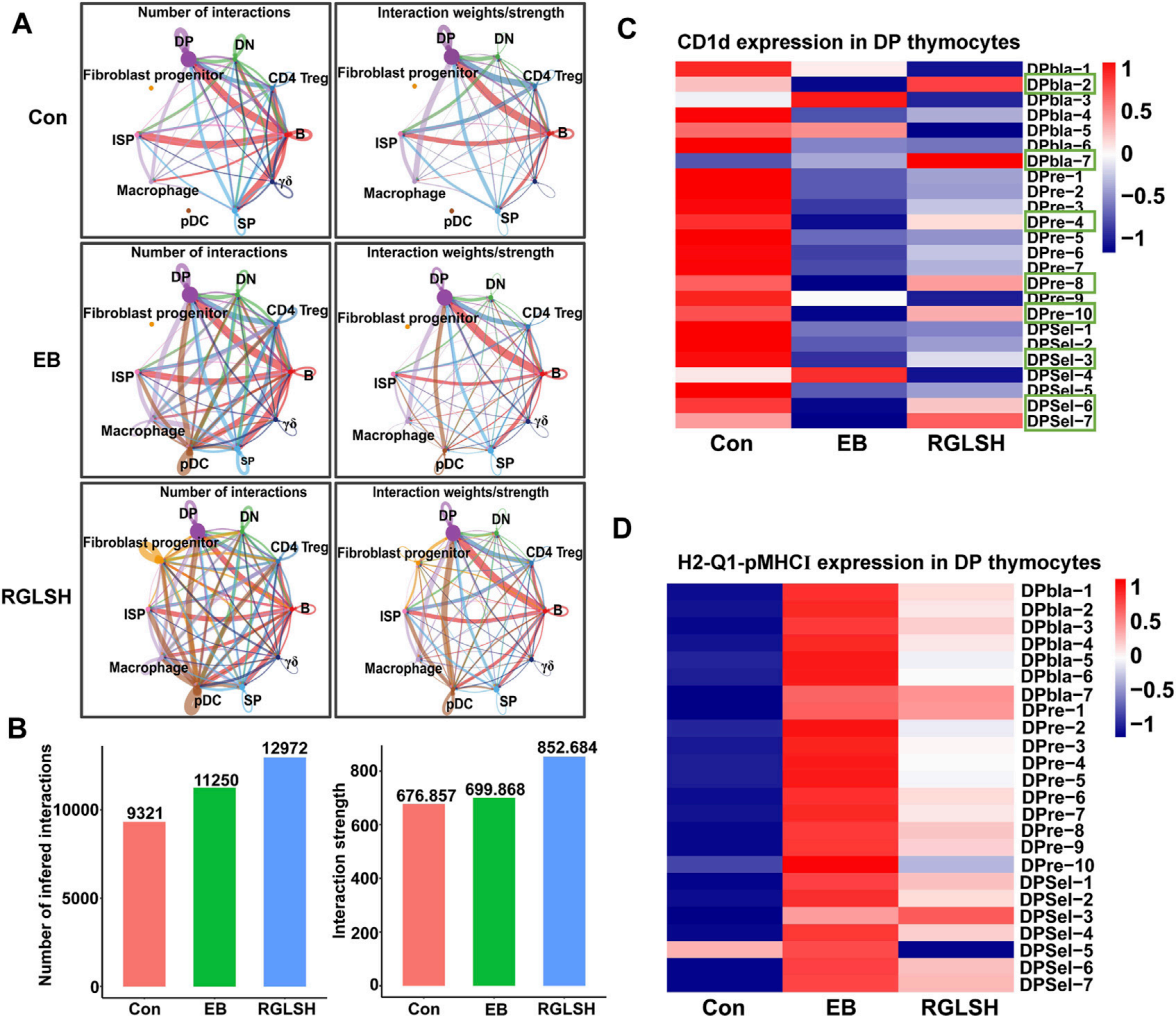
RGLSH restricts pMHC-CD8 communication between DPsel-7 thymocytes and DP thymocytes. **(A,B)** Effect of RGLSH treatment on the number and intensity of interactions between thymic stromal cells. **(C)** Effect of RGLSH treatment on the expression of CD1d in DP thymocytes. **(D)** Effect of RGLSH treatment on the expression of H2-Q1-pMHCⅠ in DP thymocytes.

In contrast to the classical mechanism by which DP thymocytes recognize pMHC for differentiation into CD4^+^ or CD8^+^ SP thymocytes, DP thymocytes undergo a distinct process when differentiating into iNKT cells specifically recognizing glycolipid antigens presented by DP thymocytes (Chandra and Kronenberg, 2015). Upon analyzing the global interactions between various cell populations following EB or RGLSH treatment (Figures 7A, B), a consistent increasing trend in total interactions, including both the number and intensity, was observed. Intriguingly, compared to the Con group, EB treatment significantly reduced the intensity of DP-DP thymocyte interactions. Conversely, RGLSH treatment, in comparison to EB, exhibited an upward trend in the intensity of DP-DP thymocyte interactions (Figure 7A). On the other hand, CD1d, a non-classical pMHC molecule expressed in DP thymocytes, serves as a key presenter of glycolipid antigens (Speak et al., 2008). Our investigation revealed varied expression levels of CD1d in DPblas, DPres, and DPsels thymocytes (Supplementary Figure S5A). Examining the impact of RGLSH on CD1d expression in DP thymocytes, we found that EB treatment notably reduced CD1d expression, whereas RGLSH treatment increased CD1d expression in 16 out of 24 DP thymocyte subgroups, including DPbla-2, DPbla-7, DPre-4, DPre-8, DPre-10, DPsel-3, DPsel-6, and DPsel-7 (Figure 7C). This suggests that RGLSH may contribute to the presentation of glycolipid antigens through enhanced CD1d expression in DP thymocytes.

**FIGURE 8:**
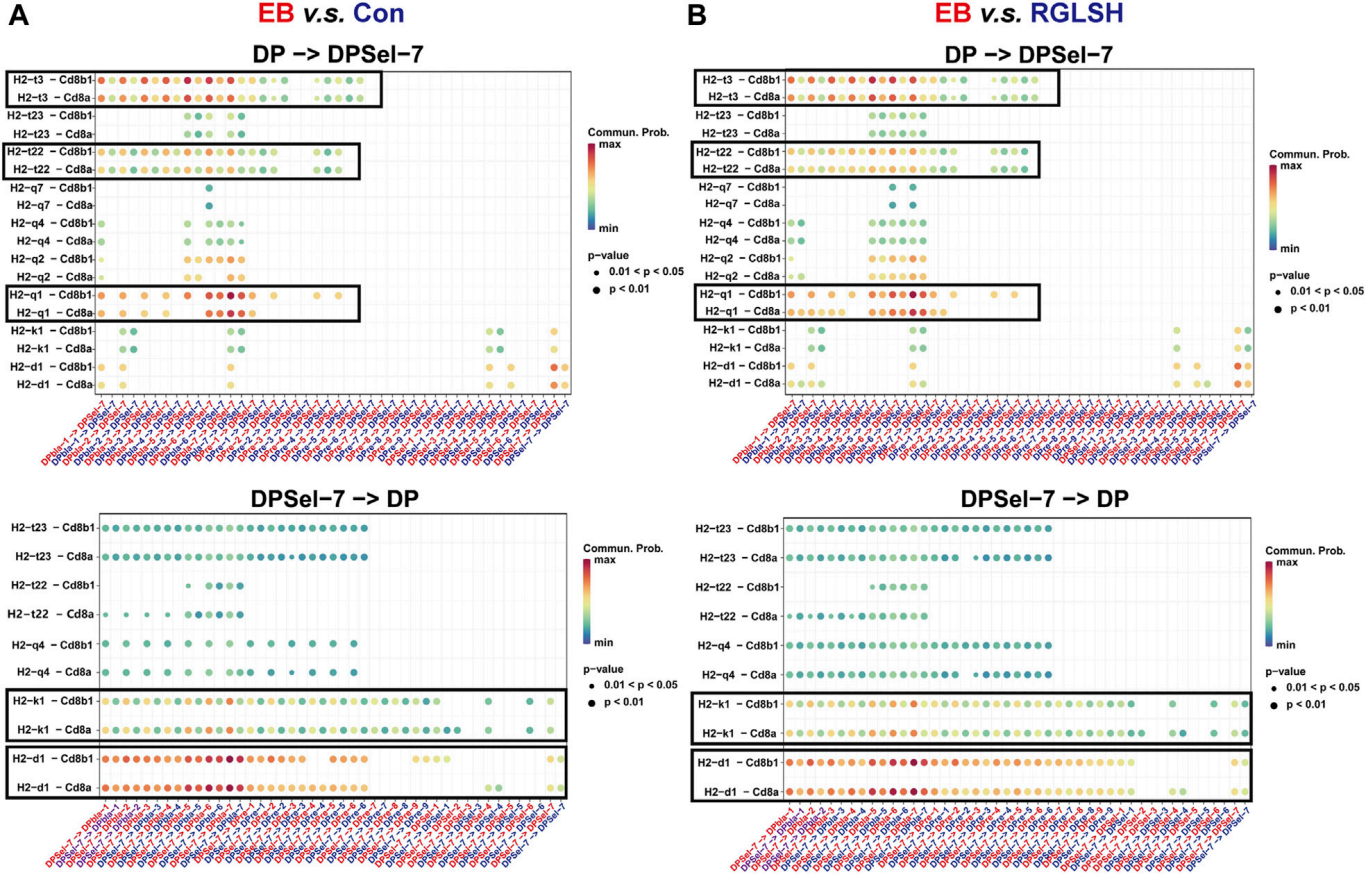
Effect of EB **(A)** and RGLSH **(B)** treatment on the ligand-receptor interaction between DPsel-7 thymocytes and DP thymocytes.

To understand how DPsel-7 is regulated by RGLSH and differentiates towards iNKT, we scrutinized the interactions between DPsel-7 thymocytes (stage 0 iNKT) and DP thymocytes. RGLSH treatment resulted in a decrease in both the number and intensity of DPsel-7 thymocytes’ interactions with DP thymocytes (Supplementary Figures S5B–D). Furthermore, we delved into the types of ligand-receptor interactions involving DPsel-7 thymocytes and DP thymocytes. Intriguingly, pMHCI-CD8 interactions between DPsel-7 thymocytes and DP thymocytes were significantly enhanced after EB treatment (Figure 8A). In contrast, RGLSH treatment mitigated this interaction (Figure 8B). Notably, the subtypes of DP thymocytes whose pMHC I-CD8 interaction with DPsel-7 thymocytes was impaired by RGLSH were highly consistent with the subtypes whose CD1d expression was enhanced by RGLSH (Figure 7C), such as DPbla-2, DPbla-7, DPre-4, DPre-8, and DPsel-7 thymocytes. Additionally, RGLSH significantly compromised EB-induced pMHC expression in DP thymocytes, which includes both pMHCⅠ (H2-K1, H2-Q1) and pMHCⅡ (H2-Aa, H2-Ab1, and H2-Eb1) moleculars (Figure 7D; Supplementary Figure S5E). We have placed particular emphasis on the H2-Q1 MHCI molecule, known for its strong effect on CD8 T cells (Figures 8A, B). Georgieve et al. reported that the expression of MHCⅠ in DP thymocytes leads to the expansion of peptide-specific PLZF^+^ innate-like (PIL) T cells, and the absence of pMHC on DP thymocytes favors the selection of non-peptides (such as CD1d and MR1), promoting iNKT cell differentiation (Georgiev et al., 2021). These results suggest that RGLSH may restrict pMHC-specific selection and enhance non-peptide-specific selection by decreasing the expression of pMHCⅠ, pMHCⅠ-CD8 interactions, and increasing the expression of CD1d in DP thymocytes, thus promoting iNKT cell differentiation.

**FIGURE 9:**
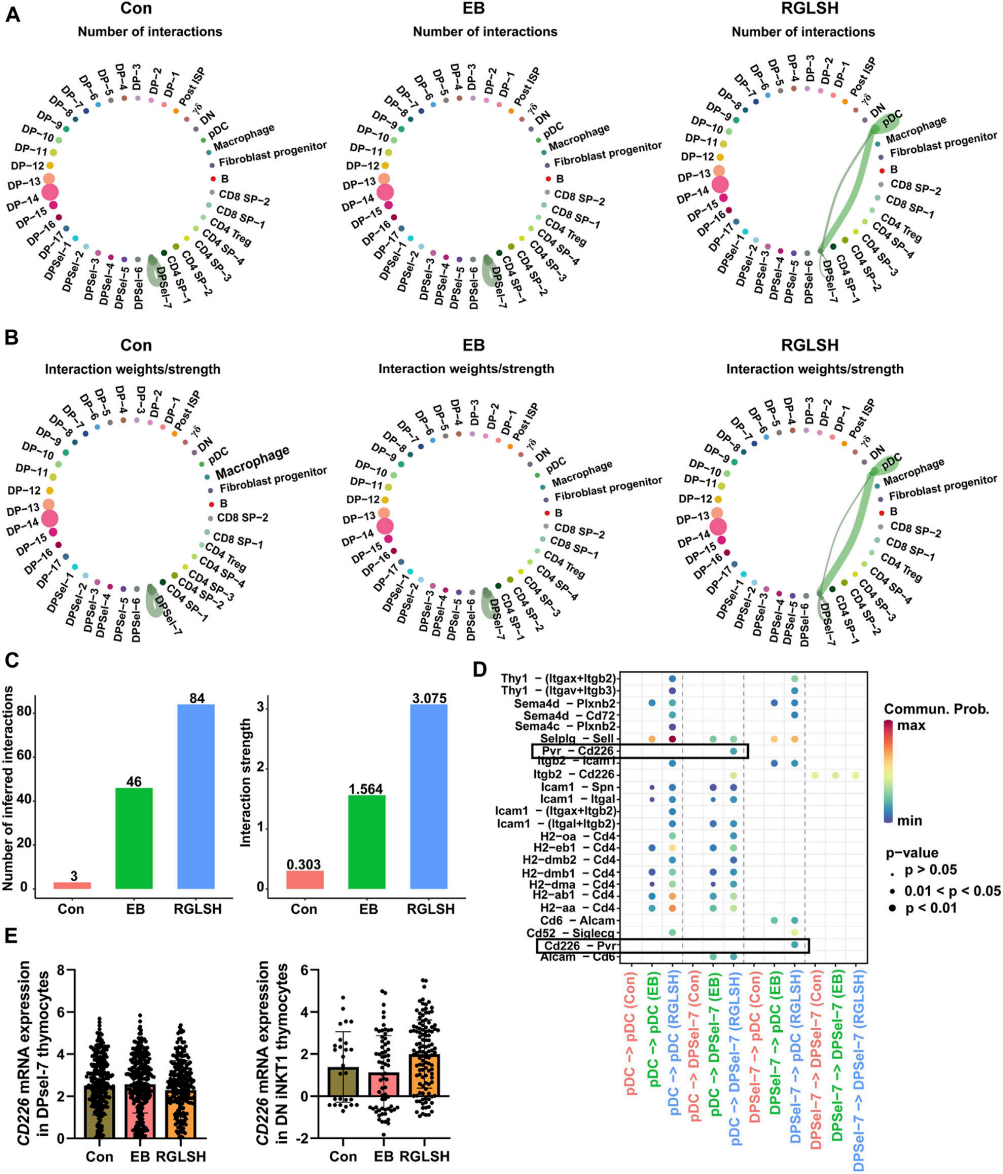
RGLSH facilitates the differentiation of DPsel-7 thymocytes into DN iNKT1 cells. **(A–C)** Effect of RGLSH treatment on the number **(A, C)** and intensity **(B,C)** of DPsel-7 thymocytes’ interactions with pDCs. **(D)** Ligand-receptor interaction analysis between DPsel-7 thymocytes and pDCs. **(E)** Effect of RGLSH treatment on the expression of *CD226* mRNA in DPsel-7 thymocytes and DN iNKT1 cells.

After TCRα rearrangement, stage 0 iNKT cells undergo a process where they first lose CD8, subsequently losing CD4 (CD4^−^CD8^−^, stage 0 DN iNKT) or retaining CD4 (CD4^+^CD8^−^, stage 0 CD4^+^ iNKT). The retained CD4 can interact with CD1d to complete the selection (Yassai et al., 2012; Oh et al., 2018). However, the mechanism by which stage 0 DN iNKT cells recognize glycolipid antigens presented by CD1d has been a subject of interest. Oh et al. (2018) found that CD8^+^ iNKT cells recognize and undergo negative selection for CD1d expressed by thymic epithelial cells (TECs) and plasmacytoid dendritic cells (pDCs), suggesting the involvement of pDCs in iNKT cell development (Keller et al., 2017). To understand the interaction between stage 0 iNKT cells and pDCs, we conducted a cell-cell communication analysis. Compared with the Con and EB groups, RGLSH treatment significantly enhanced both the frequency and intensity of interactions between stage 0 iNKT cells and pDCs (Figures 9A–C). Notably, ligand-receptor interaction analysis revealed that RGLSH increased the interaction of CD226 and Pvr (CD155) (Figure 9D) but did not affect the expression of *CD226* in stage 0 iNKT and DN iNKT cells (Figure 9E). In mice, T cell immunoreceptor with Ig and ITIM domain (TIGIT), the primary receptor for Pvr/CD155, serves as a co-inhibitory receptor for T and NK cells. The interaction between TIGIT and CD155 can directly inhibit the function of T cells and NK cells (Sarhan et al., 2016; Rotte et al., 2021). Conversely, enhancing CD226-CD155 interaction competitively inhibits CD155-TIGIT interactions (Sarhan et al., 2016). a phenomenon that may contribute to stage 0 iNKT cells acquiring NK cell properties while losing CD4^+^ and CD8^+^. This emphasizes a nuanced regulatory mechanism where RGLSH, by modulating CD226-CD155 interactions, may influence the fate and functional properties of developing iNKT cells.

## 3 Discussion

Traditional Chinese Medicines (TCMs) hold a rich history in Asian cultures, serving as integral components in preventing and treating many diseases. For decades, research has illuminated the impact of TCMs and their constituents on immune cells and cytokine production associated with immune responses (Ma et al., 2013). However, the intricate mechanisms through which TCMs modulate immune function remain elusive, posing a significant scientific challenge highlighted by the China Association for Science and Technology in 2020. Among these TCMs, Ganoderma lucidum (G. lucidum) is a rare and esteemed herb renowned for its life-prolonging attributes (Bishop et al., 2015). Accumulating evidence underscores the robust immunomodulatory functions of G. lucidum and its Ganoderma lucidum spores (GLS) (Wang and Lin, 2019; Xu and Li, 2019). Particularly noteworthy is their efficacy in enhancing T cell-mediated cellular immunity, leading to increased proportions of T cells in peripheral blood and augmented T cell activation (Wang et al., 2018; Su et al., 2021). Despite this wealth of knowledge, the specific impact, and underlying mechanisms of GLS on T cell development within thymus tissue, a crucial site for T cell differentiation, remain unexplored.

**FIGURE 10:**
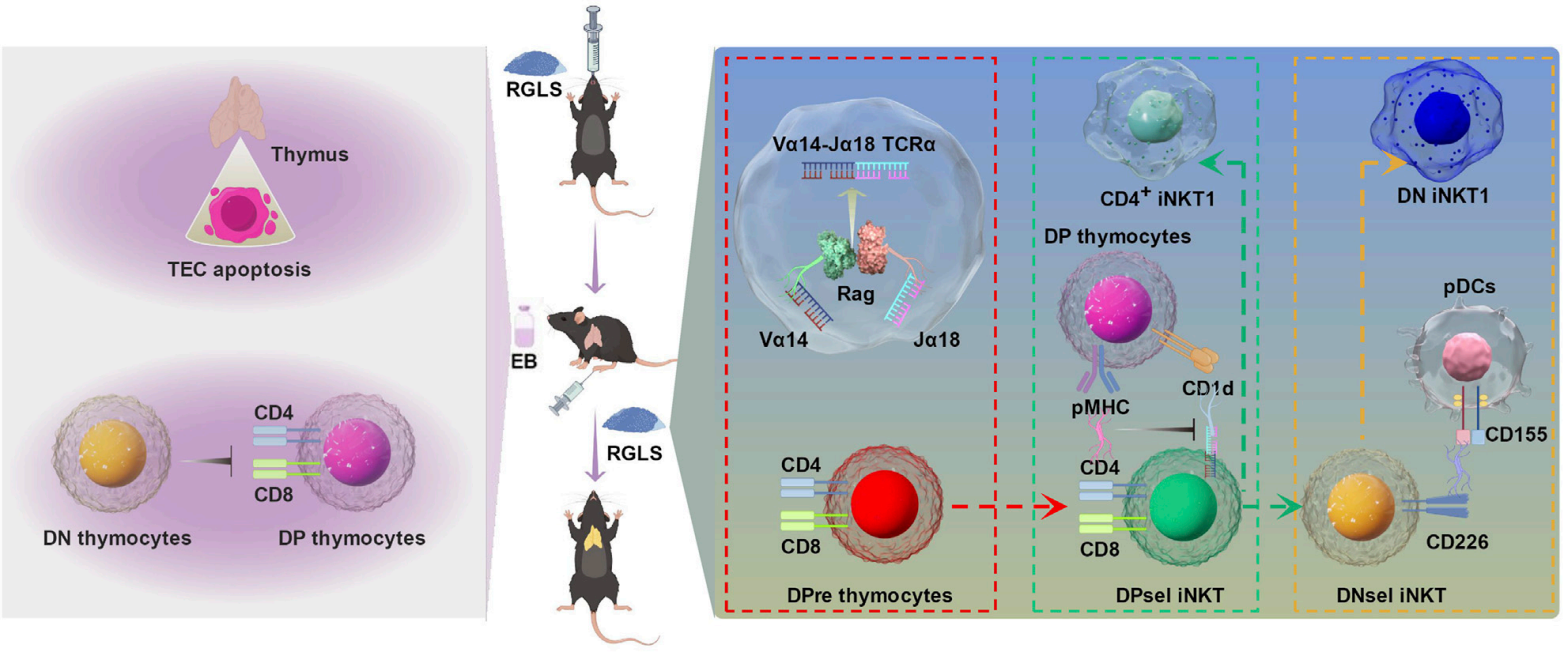
The protective effect of GLS on EB-induced thymic atrophy and T cell development and the underlying mechanism. On the one hand, GLS treatment significantly protected EB-induced thymic atrophy by inhibiting apoptosis of TECs. On the other hand, GLS facilitates thymocyte development from DN to DP stage and DPres thymocytes into iNKT cells, which was mediated by the Vα14-Jα18 TCRα rearrangement, DP-DP thymocyte interactions, and DP thymocyte-pDCs interactions.

Our study addressed this gap by simulating hormone-induced thymic atrophy using estradiol benzoate (EB), an estrogen analog. As summarized in Figure 10, our investigation into the regulatory effects of GLS on thymocyte (T cell precursor) development revealed a significant improvement in EB-induced thymic atrophy. Moreover, GLS, including RGLS and BGLS, effectively increased the proportion of CD4^+^ T cells in peripheral blood, with the superior efficacy of RGLS over BGLS. Mechanistically, GLS mitigated EB-induced apoptosis and cell cycle arrest of thymic epithelial cells (TECs), creating a conducive environment for thymocyte development. More precisely, RGLSH emerged as a key player in promoting the development of double-positive (DP) thymocytes into iNKT cells. This effect was orchestrated by enhancing the formation of TRAV11-TRAJ18 (Vα14-Jα18) T cell receptor alpha (TCRα) chains during the rearrangement phase and restricting peptide-major histocompatibility complex (pMHC)-CD8 interactions in DP thymocytes during the selection phase.

Shifting our focus to the structural elements of the thymic microenvironment, cortical thymic epithelial cells (cTECs) and medullary thymic epithelial cells (mTECs) play essential roles. cTECs regulate the positive selection of DP thymocytes by presenting pMHC-associated self-antigens, while mTECs contribute to negative selection by expressing tissue-restricted antigens (TRAs). Estrogen has been implicated in thymic atrophy through the Fas/FasL-mediated apoptosis pathway, impacting TEC function and quantity (Yao and Hou, 2004; Wang et al., 2008). Our study revealed that EB-induced thymic atrophy, marked by increased apoptotic TECs and altered cortical thickness and cTEC density, shares pathological characteristics with age-related thymic atrophy. Unlike conventional T cells, iNKT cell development appears to be more dependent on interactions between thymocytes themselves, as well as between thymocytes and antigen-presenting cells such as macrophages and dendritic cells (Chandra and Kronenberg, 2015; Cruz et al., 2022). Dendritic cells, as an important component of thymic stromal cells, participate not only in iNKT cell development but also in the negative selection process of thymocytes (Li et al., 2021a). However, thymic atrophy induced by senescence leads to a narrowing of the cortical and medullary regions, resulting in an increased density of thymic resident dendritic cells (Li et al., 2021a). This increase may affect the migration of dendritic cells within the three-dimensional network structure of the thymus, thereby reducing the probability of interaction between thymocytes and dendritic cells. Importantly, RGLSH has exhibited potential benefits in mitigating age-related thymic atrophy, which warrants further investigation.

The inevitability of thymic atrophy and injury, exacerbated by stress, infection, and aging, underscores the importance of thymic regenerative capacity. While the thymus possesses a remarkable ability for self-repair, this diminishes with age. Strengthening or restoring thymic regenerative capacity is a viable strategy to counteract thymic atrophy caused by various factors (Alawam et al., 2020). Hormone-induced thymic atrophy, characterized by apoptosis of TECs, was addressed in our study. Interestingly, RGLSH not only counteracted EB-induced thymic atrophy but also promoted the expression of cell cycle-related genes, indicating its role in enhancing TEC proliferation and contributing to thymus functional maintenance.

Moving to the intricate process of thymocyte development, our findings shed light on how RGLSH influences the transition from double-negative (DN) to mature T cells. By analyzing thymocyte subtypes and employing TCR sequencing, we observed that RGLSH promoted the development of the DN stage to the DP stage and facilitated the formation of Vα14-Jα18 TCR, consistently expressed in iNKT cells. Moreover, our research highlighted a unique development trajectory, indicating that RGLSH influenced the DPsel-1→PDsel-4→DPsel-5→DPsel-6→DPsel-7 axis, ultimately promoting the development of DPsel-7 into CD4 SP-2. The biomarker characteristics of DPsel-7 and CD4 SP-2 suggested similarities to stage 0 iNKT and iNKT cells, respectively. These findings support the notion that RGLSH regulates DP thymocyte differentiation into iNKT cells.

In contrast to humans, murine iNKT cells primarily express CD4 or neither CD4 nor CD8 (Godfrey et al., 2010). Potentially explaining the lack of influence on CD8^+^ T cells in peripheral blood by RGLSH. However, a challenge remains: it is currently unknown whether Ganoderma lucidum spores can regulate iNKT cell development in humans. It is well established that the diversity of T cell functions is contingent upon the diversity of TCRs (Gu et al., 2022). In mice, iNKT cells consistently express the Vα14-Jα18 TCRα chain, while human iNKT cells consistently express the Vα24-Jα28 TCRα chain (Gu et al., 2022). Despite their different transcriptional backgrounds, the development of iNKT cells necessitates recognition of glycolipid antigens presented by CD1d (Guo et al., 2016). Unfortunately, no studies have reported that Ganoderma lucidum spores regulate iNKT cell development in clinical or humanized animal models. Interestingly, the high conservation of classical TCR and CD1d molecules allows for cross-reactivity between different species, i.e., mouse iNKT cells can recognize human CD1d and *vice versa* (Matsuda et al., 2008; Guo et al., 2016), indicating the potential of Ganoderma lucidum spores to regulate human iNKT cell development. However, further research is required to confirm this. It is noteworthy that no Chinese medicine or natural products have been reported to promote iNKT cell development to date. If Ganoderma lucidum spores are found to influence human iNKT cell development, it would indeed mark a significant advancement in the field of immunomodulation within TCMs.

Thymocyte development involves intricate interactions between thymocytes and various thymic stromal cells, such as macrophages, dendritic cells, and thymic epithelial cells. Our exploration of DP thymocyte positive selection unveiled a unique developmental line for iNKT cells, where positive selection involves thymocyte-thymocyte interactions rather than interactions with thymic epithelial cells (Georgiev et al., 2021). Notably, RGLSH significantly reduced pMHCⅠ expression and pMHCⅠ-CD8 interaction in DP thymocytes, suggesting an enhancement of non-pMHC selection among DP thymocytes. Mechanistically, RGLSH modulated the expression of JNK2, a negative regulator of CD1-mediated glycolipid antigen presentation (Liu et al., 2019). Further supporting its role in promoting iNKT cell development.

Beyond CD4^+^ iNKT cells, our investigation delved into another subset—double-negative (DN) iNKT cells, known for their heightened ability to secrete interferon-γ (IFN-γ) (O'Reilly et al., 2011). While the development of DN iNKT cells is believed to be CD1d-dependent, the precise mechanism by which these cells recognize glycolipid antigens presented by CD1d remains unknown. Building on the theory proposed for the positive selection of stage 0 iNKT cells, our study implicates plasmacytoid dendritic cells (pDCs) in the iNKT cell development. Analyzing the interaction between DPsel-7 (stage 0 iNKT) and pDCs, we observed that RGLSH significantly enhances the interaction between CD226 and CD155. CD226, a member of the immunoglobulin superfamily, displayed a gradual increase in expression from the DP phase to the single-positive (SP) phase. Previous research has linked CD226 to crucial roles in T cell development, including promoting DP thymocyte survival by inhibiting apoptosis through Akt phosphorylation (Ma et al., 2023). Moreover, CD226 is highly expressed in terminally differentiated T cells, including natural killer T (NKT) cells. This suggests its involvement in the positive selection process and further development of DP iNKT cells (Ma et al., 2023). Notably, human iNKT cells have been observed to recognize target cells, such as leukemia cells (CD1d^−^), in a Vα24-Vβ11 T cell receptor (TCR)-mediated manner, raising questions about whether murine DPsel iNKT cells can recognize DP thymocytes via Vα14-Vβ8.2/Vβ7/Vβ2 TCR (equivalent to Vα14-Vβ11 TCR in human iNKT cells) and complete positive selection. This intriguing avenue deserves further investigation to elucidate the potential parallels between human and murine iNKT cell development. Adding another layer of complexity, studies have highlighted the role of CD226 in driving CD4^+^ T cells toward differentiation into Th1 helper cells (Shibuya et al., 2003; Yamashita-Kanemaru et al., 2015). Coincidentally, DN iNKT cells exhibit stronger Th1 properties than CD4^+^ iNKT cells (O'Reilly et al., 2011). Loss of CD226 or CD155 has been associated with an increase in the frequency and number of iNKT1 cells (Th1, secreting IFN-γ and IL-4, NK1.1^+^) at the expense of iNKT2 (Th2, secreting IL-4, NK1.1^−^) and iNKT17 (Th17, secreting IL-17a, NK1.1^−^) cells. This suggests a potential compensatory mechanism for RGLSH in the iNKT1 cell differentiation might through CD226 and CD155 as a co-activator of Th1-like cell differentiation to form DN iNKT cells and iNKT1 cells, thus being independent of CD1d.

## 4 Materials and methods

### 4.1 EB-induced thymic atrophy model

Eight-to-ten-week (18–20 g) adult male ICR mice were purchased from Hangzhou Medical College (animal quality certificate number: 2003200027) and kept in a specific-pathogen-free (SPF) environment. The experiment was conducted under the “Principles of Laboratory Animal Care Committee of National Institutes of Health (NIH)” and all animal experiments were approved by the Animal Ethics Committee of Zhejiang Research Institute of Traditional Chinese Medicine (Approved Number: 20190002). All procedures of the experiment are following the Animal Welfare Act Regulations. After 2 weeks of adaptation, the ICR mice (n = 109) were randomly divided by weight into seven groups: Con group (saline, n = 16), EB group (0.1 mg/day, n = 16), EB plus Ubenimex group (Ube, 5 mg/kg, n = 16), EB plus high-dose BGLS treatment (BGLSH, 1.4 g/kg/day, n = 16) group, EB plus low-dose BGLS treatment (BGLSL, 0.7 g/kg/day, n = 15) group, EB plus high-dose RGLS treatment (RGLSH, 1.4 g/kg/day, n = 15) group, and EB plus low-dose RGLS treatment (RGLSL, 0.7 g/kg/day, n = 15) group. In studies of RGLSH regulating T cell development, the mice (n = 48) were randomly divided by weight into three groups: Con group (treatment with saline, n = 16), EB group (0.1 mg per time, n = 16), and EB plus high-dose RGLS treatment (RGLSH, 1.4 g/kg, n = 16) group. Except for the Con group and EB group, the other groups were administered with Ube, BGLS, or RGLS for 14 days (gavage, quaque die), and on the fourth day of execution, except for the Con group, the other groups were administered with EB for 6 times (intraperitoneal injection, 0.1 mg/day, quaque omni die). Body weights were measured on day 1, day 7, and day 15, and the animals were euthanized. For further analyses, the blood and thymus tissue were collected, weighed, and stored at −80°C.

### 4.2 WBC analysis

The 150 μL of EDTA-K2-treated anti-coagulant peripheral blood were collected, and WBC classification and proportion were measured using an XT-2000i fully automatic hematology analyzer system (Sysmex Shanghai Co., Ltd. Shanghai, China).

### 4.3 Thymus tissue histological examination

The harvested thymus tissues were fixed in 4% paraformaldehyde, embedded in paraffin, and cut into 5-μm sections. Then, the sections were stained with hematoxylin and eosin (H and E) according to the manufacturer’s protocol. The morphological changes in thymus tissues were observed, and images were randomly captured by a microscope (DM4000, Leica Biosystems).

### 4.4 TUNEL staining

Apoptosis of TECs was determined using TUNEL staining. Thymus tissue samples were harvested and fixed in 10% formalin for 24 h. Fixed thymus tissues were embedded in paraffin, and 5-μm sections were cut and used for TUNEL staining using the TUNEL assay kit (Beyotime Biotechnology, Cat: C1098) according to the manufacturer’s instructions.

### 4.5 Flow cytometric analysis of T cell subtypes and proportions

The 100 μL of EDTA-K2-treated anti-coagulant peripheral blood was collected, and 2 μL of CD3, CD8, and CD8 fluorescent antibodies were added successively, and incubated at room temperature (RT) for 15 min in the dark. Then add 1 mL erythrocyte lysate and incubate at RT for 15 min in the dark, centrifuge (500 g, 5 min) and discard the supernatant. Next, add 1 mL sterile PBS to wash precipitation, centrifuge (500 g, 5 min), and discard supernatant. Finally, 500 μL sterile PBS was added to resuspend precipitation, and the proportion of CD4^+^ and CD8^+^ T cells was measured using flow cytometry. To detect the T cell subtype and proportion in the thymus, the thymus tissues were cut into about 1 mm^3^ tissue blocks and repeatedly washed with sterile PBS until the PBS was clear. PBS was mixed, the tissue blocks were removed through a 40 μm filter membrane, and centrifuge (500 g, 5 min) to discard the supernatant. Next, add 1 mL sterile PBS to resuspend precipitation, and centrifuge (500 g, 5 min) to discard supernatant. Finally, 500 μL sterile PBS was added and 100 μL suspension was taken to incubate with 2 μL of CD3, CD4, and CD8 fluorescent antibodies at RT for 15 min in the dark. The proportion of CD4^−^CD8^−^, CD4^−^CD8^+^, CD4^+^CD8^−^, and CD4^+^CD8^+^ thymocytes was measured using flow cytometry.

### 4.6 RNA isolation and qRT-PCR

About 30 μg of thymus tissue was pulverized using liquid nitrogen cryogenic freezing, and 500 μL of lysate was added to fully split the tissue, the total RNA was extracted using an EASYspin reagent kit (Biomed biotech, Beijing, China, Cat: RA105-01) according to the manufacturer’s protocol. Total RNA quality and quantity were analyzed using a NanoDropTM one spectrophotometer (Thermo Scientific, Grand Island, NY, United States). An iScript Reverse transcription supermix (Vazyme Biotech, Nanjing, China) was used to synthesize first-strand cDNA from 1 μg total RNA. qRT-PCR analysis was performed using Faststart Essential DNA Green Master (Roche, Basel, Swit, Cat: 06402712001) on LightCycler96 Real-Time PCR system (Roche). Primers were designed with Primer3Plus software (Cambridge, MA, United States). GAPDH was used as the reference gene. The expression level of mRNA was calculated according to the formula: ΔCt = Ct (sample)−Ct (GAPDH), ΔΔCt (sample) = ΔCt (sample)−ΔCt (calibrator). The fold change of mRNA was calculated using the relative quantification (2^−ΔΔCT^). Primer sequences of qRT-PCR reactions is as follows: *pMHCⅡ*, F: TTT​GAA​GCA​TGG​CAC​GTT​GG, R: CAC​CTC​AGG​GTG​ACA​TTC​CC. *GAPDH*, F: TGA​ACG​GGA​AGC​TCA​CTG​G, R: GAG​CTT​CAC​AAA​GTT​GTC​ATT​GAG.

### 4.7 Label-free proteomic analysis

After the washout of thymocytes, the total protein of thymus tissue was extracted, enzymatically digested, and desalted before separation using nanoliter reversed-phase liquid chromatography and detection by mass spectrometry. Protein abundance was quantified using a Proteome Discoverer library search. Before performing differential protein expression analysis, we used the mice (v3.16) package to perform multiple imputations and fill in missing values. Next, we took the logarithm of the protein expression matrix. We then used the edgeR (v3.42) package to identify differential proteins with adjusted *p*-values less than 0.05. Finally, we conducted functional enrichment using the clusterProfiler (v4.8) package.

### 4.8 TCRα sequencing

Total RNA was isolated from the thawed mononuclear cells from the grafts or from the peripheral blood of the recipients using the RNeasy Plus Mini Kit (Qiagen). RNA samples were analyzed by NGS for TRAs using the ImmuHub^®^ TCR profiling system (ImmuQuad Biotech, Hangzhou, China). Briefly, a 5′ RACE unbiased amplification protocol was used. One common forward adaptor primer and one reverse primer corresponding to the constant (C) regions of each of the TCRα were designed to facilitate PCR amplification of cDNA sequences in a less biased manner. This protocol uses unique molecular identifiers (UMIs) introduced during cDNA synthesis to control bottlenecks and eliminate PCR and sequencing errors. Sequencing was performed on an Illumina NovaSeq^®^ system with PE150 mode. The UMI attached to each raw sequence read was applied to correct PCR and sequencing errors correction and PCR duplicate removal. Map V, D, J, and C segments with NCBI^®^ and then extract CDR3 regions and assemble clonotype for all clones. The resulting nucleotide and amino acid sequences of CDR3 of TCRα were determined and those with out-of-frame and stop codon sequences were removed from the identified TCRα repertoire. We further defined amounts of each TCRα clonotype by adding numbers of TCRα clones sharing the same nucleotide sequence of CDR3. TCRα clonotype data were processed using custom scripts in R for further analysis.

### 4.9 Single-cell RNA sequencing

Cell Ranger software (version 3.0), provided by 10× Genomics, was used to demultiplex cellular barcodes, and map reads to the genome and transcriptome using the STAR aligner to produce a matrix of gene counts versus cells. The quality control is using Seruat version 4.3, each sample was considered for genes shared by 10 cells, and cells showing 200 or more features and fewer than 5,000 features. Cells with mitochondrial RNA percentages of >10 were filtered out. Doublets were identified and removed using DoubletFinder v2.0.3 (McGinnis et al., 2019) using the recommended parameter settings. After stringent QC filtering, 50508 cells remained for downstream analysis. SCtransform normalization was performed on each sample separately, along with regression of mitochondrial RNA as a variable. Subsequently, PrepSCTIntegration was run to select features for downstream integration and FindIntegrationAnchors to identify anchor genes. The integrated data were scaled, and principal component analysis was performed. Data were visualized using Uniform Manifold Approximation and Projection (UMAP). Cell clusters were identified by a shared nearest-neighbor (SNN) modularity optimization-based clustering algorithm set at a resolution of 0.8.

### 4.10 Analysis of thymocytes developmental trajectory based on Monocle2

Monocle2 is an R package used to discover the evolutionary trajectories between cells (Qiu et al., 2017). We use the new Cell Data Set function to create a Cell Data Set object, which is used to store expression matrices, cell annotations, and gene annotations. Standardization factors and dispersion of cells and genes were calculated using the estimate Size Factors and estimate Dispersions functions. Unsupervised clustering of cells is performed using the cluster Cells function, followed by dimensionality reduction and sorting of cells using the reduce Dimensional and order Cells functions to learn the developmental trajectory and pseudo time of cells. Monocle2 uses the DDRTree algorithm to construct a main graph that reflects the similarity of cell states and automatically recognizes branch structures based on the main graph.

### 4.11 Analysis of thymocyte developmental trajectory based on Monocle3

Monocle3 is an R language-based analysis toolkit that performs cell clustering, differential expression, and pseudo-time analysis (Cao et al., 2019). We quality-controlled and normalized the raw count matrices, and then downscaled and visualized the cells using the UMAP algorithm. Then we learned the development trajectories of the cells from the reduced dimensional space using the L1-graph or spanning tree algorithm.

### 4.12 CellChat analysis

CellChat is an open-source R package (https://github.com/sqjin/CellChat) to infer, visualize and analyze intercellular communications from scRNA-seq data (Jin et al., 2021). We first group cells based on cell type labels and use Cell Chat DB. Mouse to import them into the mouse ligand-receptor database. Then, we run the Cell Chat function to calculate the communication probability between each pair of cell populations, and use net Analysis_compute Centrality function to evaluate the importance of each cell population and signaling pathway in the network. Finally, we use net Visual_aggregate function to visualize the entire intercellular communication network, as well as net Visual_diffInteraction function to compare network changes under different conditions. In order to analyze the communication differences of specific ligand-receptor pairs between DP thymocytes and DPSel-7 thymocytes, the net Visual function of R packet CellChat was used, and the communication intensity was displayed by probability difference bubble map. The color of the bubble indicates the possibility of communication, the size of the bubble indicates significance, and bubbles with a P-value of less than 0.05 were shown.

### 4.13 Co-localized immunohistochemical analysis of thymus tissues

The immunohistochemistry (IHC) analysis was referred to previous reports (Li et al., 2023). In briefly, formalin-fixed thymus tissues were embedded in paraffin and cut into 4-μm sections consecutively, and 2 adjacent sections were selected for co-localized IHC analysis of cytokeratin5 (CK5, Proteintech, Cat: 19677-1-AP)-Caspase 3 (Casp3, Proteintech, Cat: 19677-1-AP), cytokeratin8 (CK8, Proteintech, Cat: 17514-1-AP)-Casp3, and CK5-Aire (Proteintech, Cat: 22517-1-AP). The sections were deparaffinized using the citric acid buffer and incubated for 10 min at 100°C. The slides were treated with 3% hydrogen peroxide to block endogenous peroxidase activity and then incubated with 2% bovine serum albumin (BSA) for 30 min. Next, the slides were incubated overnight at 4°C. with obvious anti-mouse primary antibodies. Then, the slides were then incubated with anti-goat IgG secondary antiboty (diagbio, Cat No: db10002) for 60 min at RT. After washing, slides were stained with 3,3-diaminobenzidine (DAB), washed and counterstained with hematoxylin, dehydrated, and then mounted with a coverslip. The images were randomly captured by a microscope (DM4000, Leica Biosystems).

### 4.14 Data analysis

All data are expressed as mean ± standard error (SE). The results presented are representatives from at least three independent experiments. The student *t*-test was used for comparing two conditions, and one-way analysis of variance (ANOVA) was used for multiple comparisons. All analyses were performed using GraphPad Prism 8.0 (Graphpad Software, Inc., United States), and a value of *P* < 0.05 was considered statistically significant.

## Data Availability

The data presented in the study are deposited in the GEO repository, accession number GSE253901.
