# Supplementary material for: Protective effects of Ganoderma lucidum spores on estradiol benzoate-induced TEC apoptosis and compromised double-positive thymocyte development
Source: Front Pharmacol. 2024 Aug 16;15:1419881. doi: 10.3389/fphar.2024.1419881 (PMC11361955; doi:10.3389/fphar.2024.1419881)
Supplement: Supplementary file 1 [file DataSheet1.pdf]

Supplementary Material

1 Supplementary Figures and Tables

1.1 Supplementary Tables

Supplementary Table 1. Effect of RGLS on TCRα chain rearrangement in DPres thymocytes, the detailed information is provided in Supplementary Table 1. XLSX

1.2 Supplementary Figures

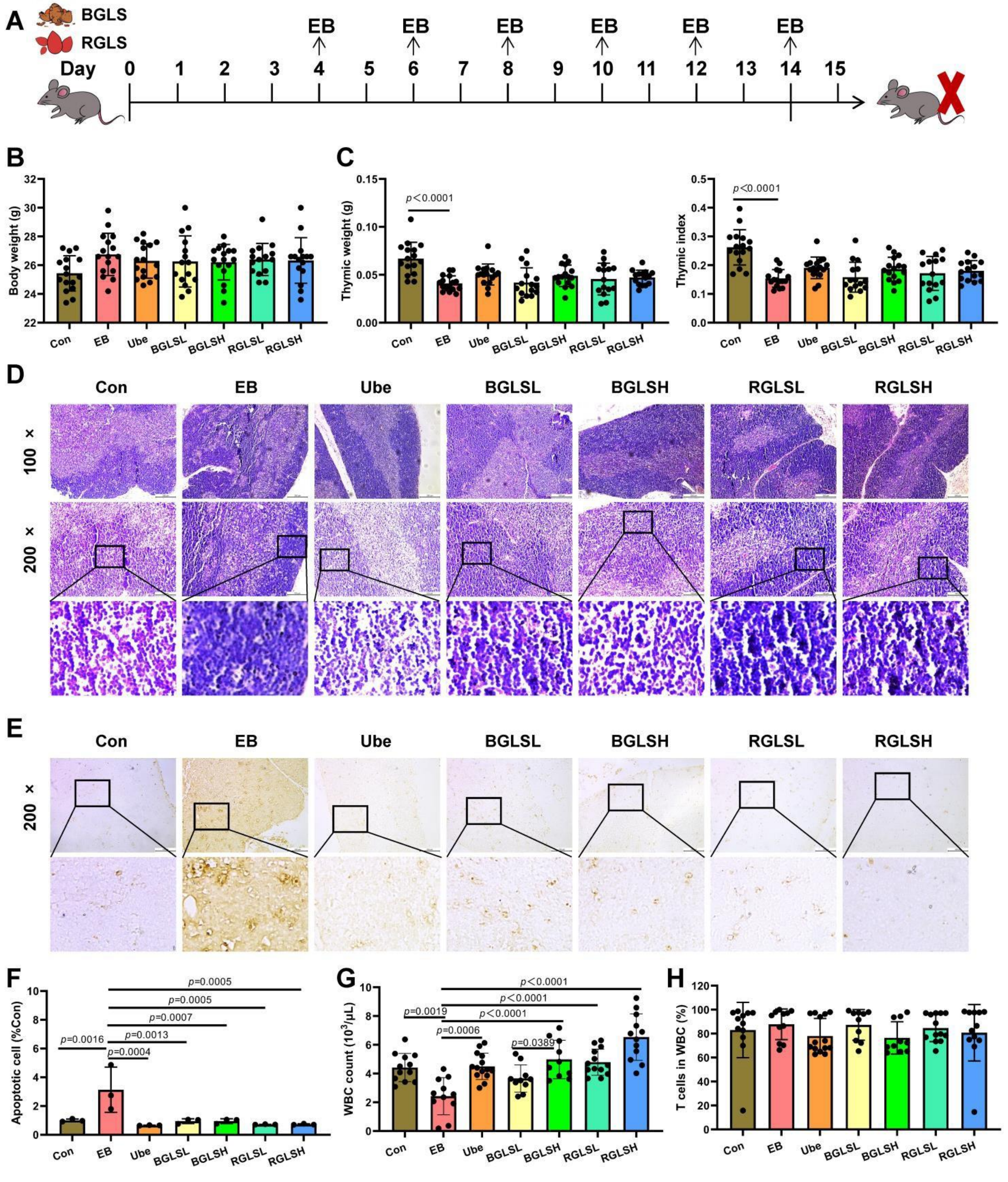

Supplementary Figure 1. GLS alleviates EB-induced thymic atrophy in vivo. (**A**) Schematic diagram of the experimental schedule. (**B**, **C**) body weight of mice (**B**), thymic weight and thymic index (**C**) of each group. (**D**) Histological analysis of thymus tissue sections was stained by hematoxylin and eosin (H&E staining). Scale bar = 200 μm (100 ×), 100 μm (200 ×), n = 3. (**E**, **F**) Cell apoptosis was detected by Tunel staining and quantitative analysis (200 ×). Scale bar = 100 μm,

n = 3. (**G**) Analysis of WBC count in peripheral blood (*n* = 10 ~ 14). (**H**) Analysis of the proportion of T cells to WBC (*n* = 10 ~ 14).

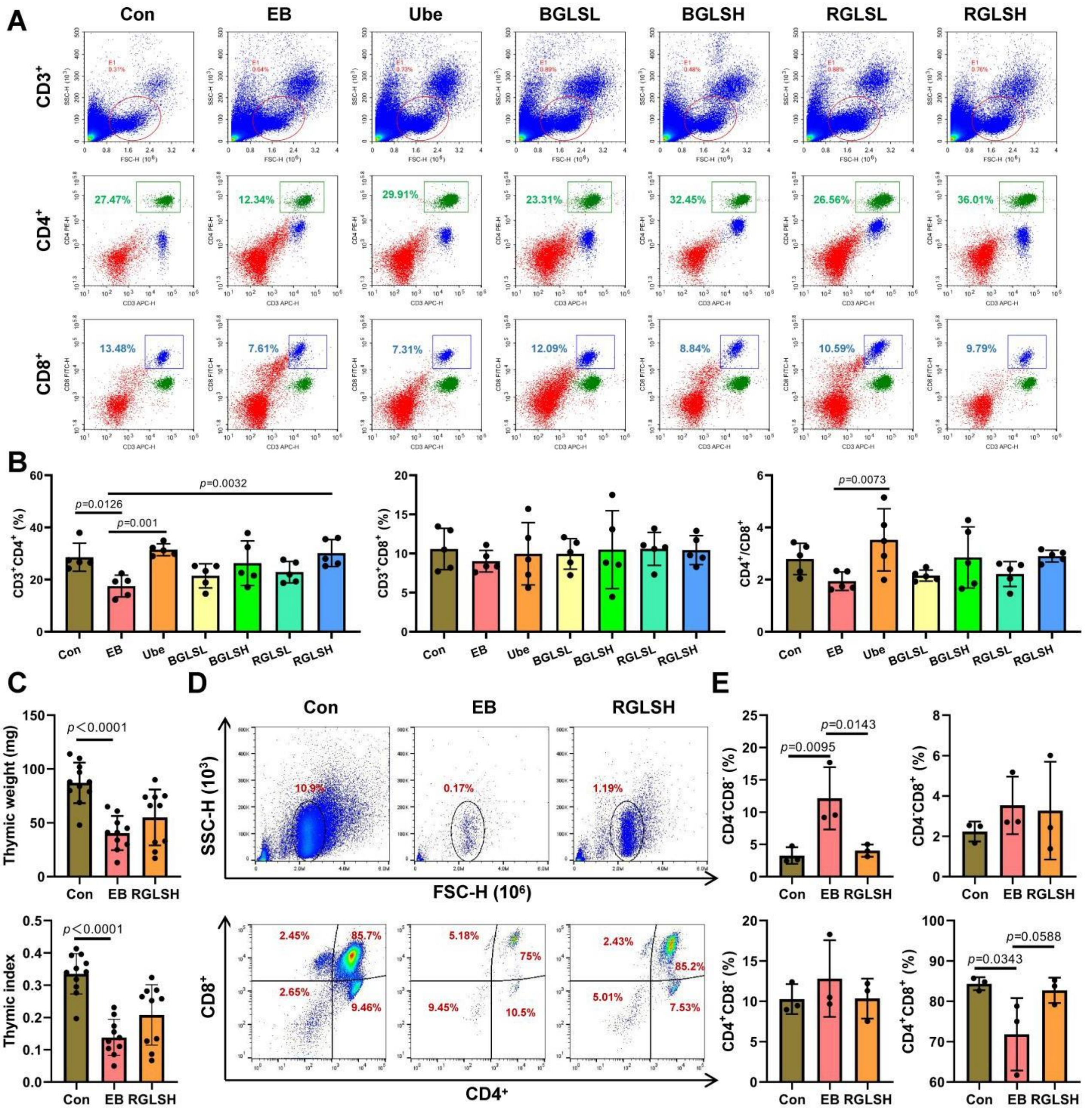

Supplementary Figure 2. RGLSH promotes T cell development in vivo. (**A**, **B**) T cell subtypes and proportions in peripheral blood were detected by flow cytometry (**A**) and quantitative analysis (**B**) (*n* = 5). (**C**) Thymic weight and thymic index of each group. (**D**, **E**) Thymocyte subtypes and

proportions in thymus tissue were detected by flow cytometry (**D**) and quantitative analysis (**E**) (n = 3).

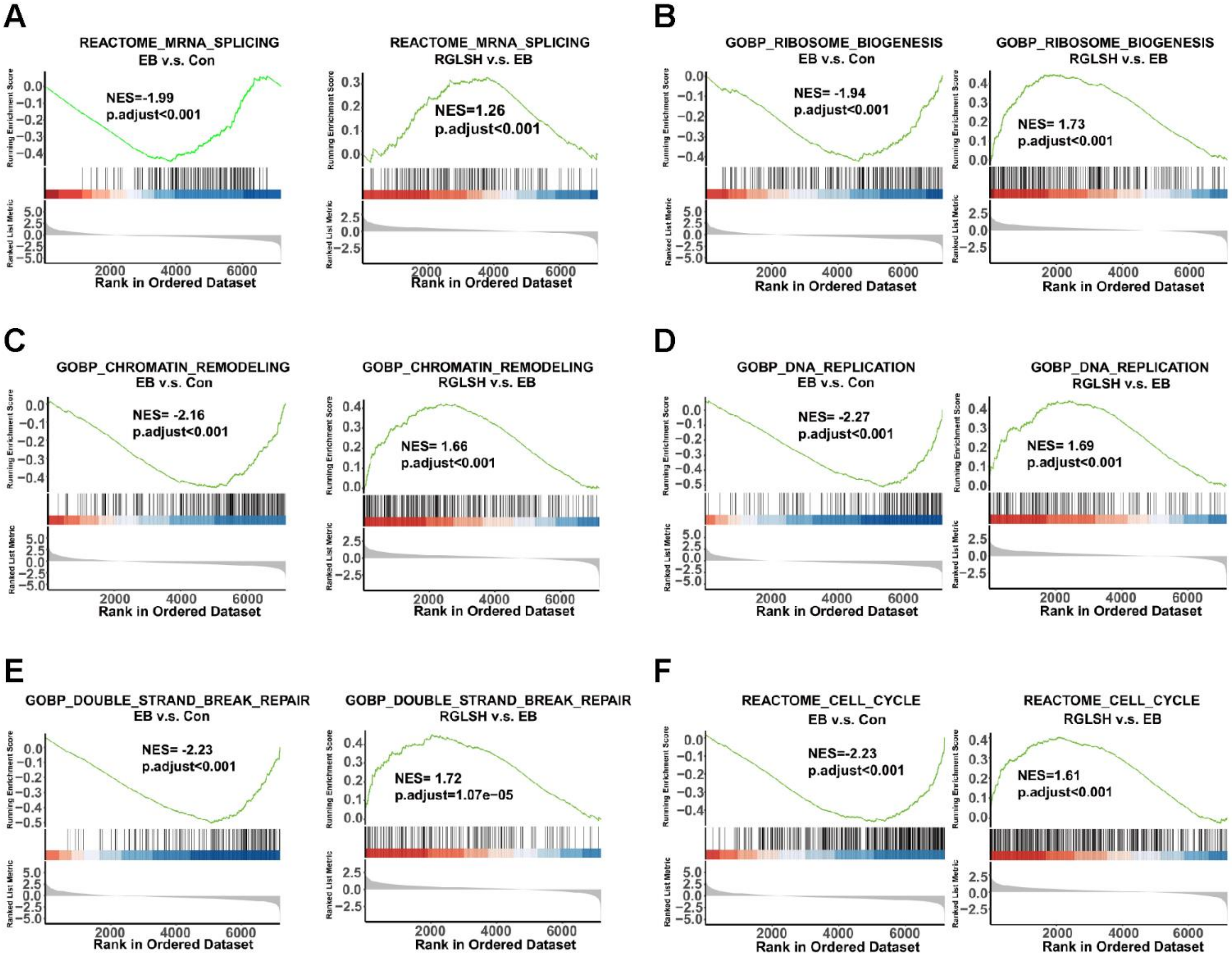

Supplementary Figure 3. Gene set enrichment analysis (GSEA) enriched by differentially expressed proteins. (**A**) mRNA splicing. (**B**) Ribosome biogenesis. (**C**) Chromatin remodeling. (**D**) DNA replication. (**E**) Double-strand break repair. (**F**) Cell cycle.

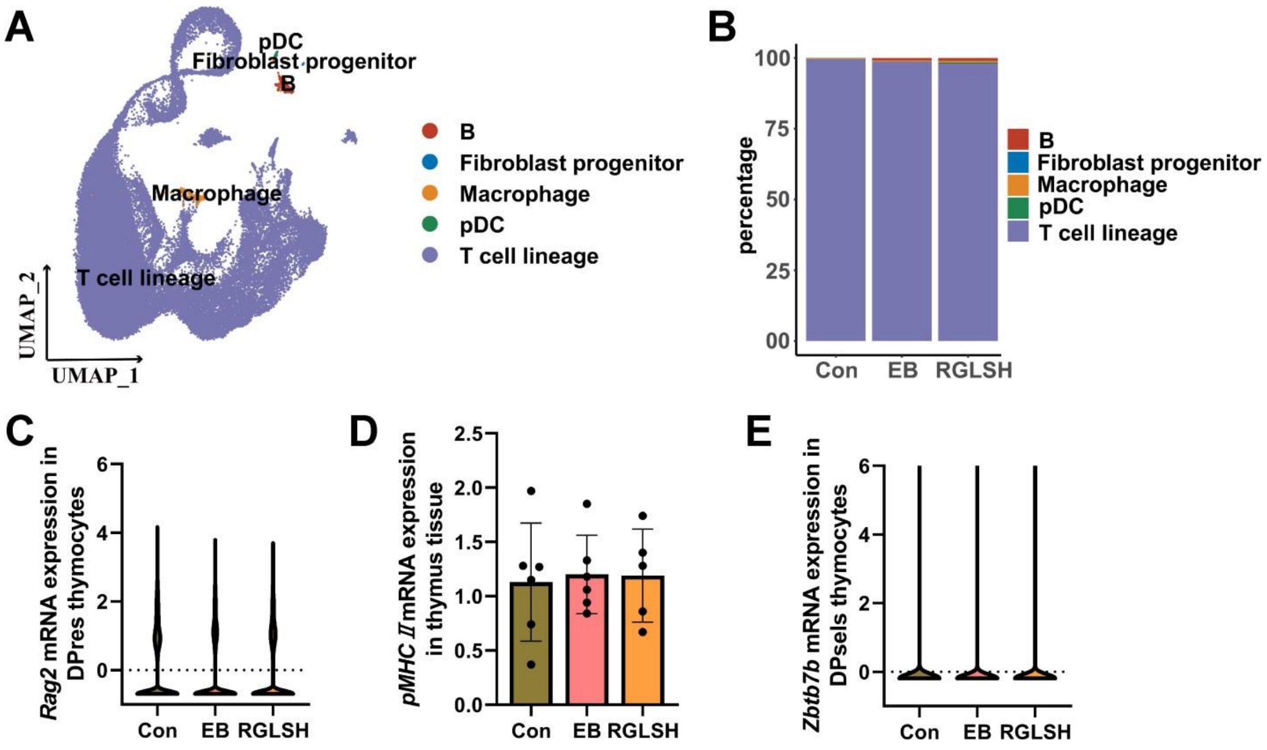

Supplementary Figure 4. RGLSH enhances DP thymocyte development and selection. (**A**) Cluster analysis of thymic stromal cells and visualization in a two-dimensional UMAP. (**B**) Proportion analysis of thymic stromal cells. (**C**) Effect of RGLSH treatment on the expression of *Rag2* mRNA in DPres thymocytes. (**D**) Effect of RGLSH treatment on the expression of *pMHCII* mRNA in

thymus tissue. (**E**) Effect of RGLSH treatment on the expression of *Zbtb7b* mRNA in DPsels thymocytes.

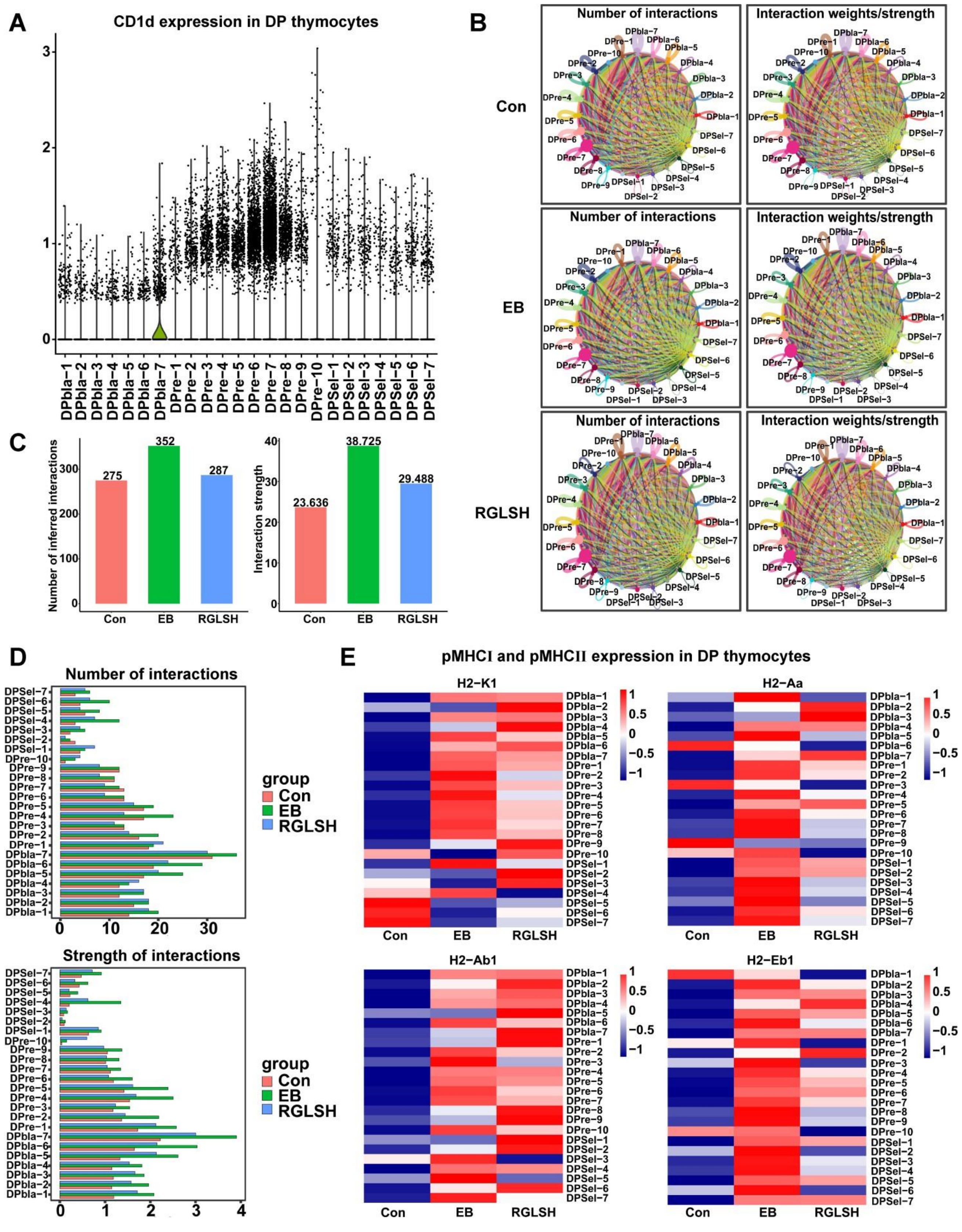

Supplementary Figure 5. RGLSH restricts pMHC-CD8 communication between DP thymocytes. (**A**) CD1d expression in DP thymocytes. (**B**-**D**) Effect of RGLSH treatment on the number and

intensity of DPsel-7 thymocytes' interactions with DP thymocytes. (**E**) Effect of RGLSH treatment on the expression of pMHCI (H2-K1) and pMHCII (H2-Aa, H2-Ab1, and H2-Eb1) in DP thymocytes.
